# A G-Protein-Coupled Receptor Modulates Gametogenesis via PKG-Mediated Signaling Cascade in Plasmodium berghei

**DOI:** 10.1128/spectrum.00150-22

**Published:** 2022-04-11

**Authors:** Peng-peng Wang, Xuefeng Jiang, Liying Zhu, Dan Zhou, Mingyang Hong, Lu He, Lumeng Chen, Shijie Yao, Yan Zhao, Guang Chen, Chengqi Wang, Liwang Cui, Yaming Cao, Xiaotong Zhu

**Affiliations:** a Department of Immunology, College of Basic Medical Sciences, China Medical University, Shenyang, China; b Department of Laboratory Medicine, Shengjing Hospital of China Medical University, Shenyang, China; c Department of Basic Medical Sciences, Taizhou University Hospital, Taizhou University, Taizhou, China; d Department of Internal Medicine, Morsani College of Medicine, University of South Florida, Tampa, Florida, USA; Hebrew University of Jerusalem

**Keywords:** malaria, G-protein-coupled receptor, gametogenesis, signal transduction, transmission-blocking activity

## Abstract

Gametogenesis is essential for malaria parasite transmission, but the molecular mechanism of this process remains to be refined. Here, we identified a G-protein-coupled receptor 180 (GPR180) that plays a critical role in signal transduction during gametogenesis in *Plasmodium.* The P. berghei GPR180 was predominantly expressed in gametocytes and ookinetes and associated with the plasma membrane in female gametes and ookinetes. Knockout of *pbgpr180* (Δ*pbgpr180*) had no noticeable effect on blood-stage development but impaired gamete formation and reduced transmission of the parasites to mosquitoes. Transcriptome analysis revealed that a large proportion of the dysregulated genes in the Δ*pbgpr180* gametocytes had assigned functions in cyclic nucleotide signal transduction. In the Δ*pbgpr180* gametocytes, the intracellular cGMP level was significantly reduced, and the cytosolic Ca^2+^ mobilization showed a delay and a reduction in the magnitude during gametocyte activation. These results suggest that PbGPR180 functions upstream of the cGMP-protein kinase G-Ca^2+^ signaling pathway. In line with this functional prediction, the PbGPR180 protein was found to interact with several transmembrane transporter proteins and the small GTPase Rab6 in activated gametocytes. Allele replacement of *pbgpr180* with the P. vivax ortholog *pvgpr180* showed equal competence of the transgenic parasite in sexual development, suggesting functional conservation of this gene in *Plasmodium* spp. Furthermore, an anti-PbGPR180 monoclonal antibody and the anti-PvGPR180 serum possessed robust transmission-blocking activities. These results indicate that GPR180 is involved in signal transduction during gametogenesis in malaria parasites and is a promising target for blocking parasite transmission.

**IMPORTANCE** Environmental changes from humans to mosquitoes activate gametogenesis of the malaria parasite, an obligative process for parasite transmission, but how the signals are relayed remains poorly understood. Here, we show the identification of a *Plasmodium* G-protein-coupled receptor, GPR180, and the characterization of its function in gametogenesis. In P. berghei, GPR180 is dispensable for asexual development and gametocytogenesis, but its deletion impairs gametogenesis and reduces transmission to mosquitoes. GPR180 appears to function upstream of the cGMP-protein kinase G-Ca^2+^ signaling pathway and is required for the maximum activity of this pathway. Genetic complementation shows that the GPR180 ortholog from the human malaria parasite P. vivax was fully functional in P. berghei, indicating functional conservation of GPR180 in *Plasmodium* spp. With predominant expression and membrane association of GPR180 in sexual stages, GPR180 is a promising target for blocking transmission, and antibodies against GPR180 possess robust transmission-blocking activities.

## INTRODUCTION

Malaria still caused an estimated 229 million cases worldwide in 2019, resulting in 409,000 deaths (1). The malaria parasite *Plasmodium* has a complicated life cycle, involving multiple morphologically distinct developmental stages in humans and mosquito vectors. During its journey through the two hosts, the *Plasmodium* parasite is exposed to host environments, sometimes so drastically different, requiring rapid adaptive responses to survive. Although signal perception and transduction are conserved in malaria parasites as in model eukaryotes, the receptors or transporters sensing the environmental triggers are different and remain poorly understood.

Heptahelical serpentine receptors are the largest group of membrane receptors responsible for transducing extracellular signals to various downstream effectors (2, 3). The serpentine receptors coupled to heterotrimeric guanine nucleotide-binding proteins belong to G-protein-coupled receptors (GPCRs) with a salient feature of seven transmembrane domains, each consisting of 25–35 residues. Noteworthy, GPCRs control major biological and pathological processes in the neural, cardiovascular, immune, and endocrine systems and are targets of approximately 40% of approved drugs currently in use (4, 5). Although there is little conservation in amino acid (aa) sequences across the entire GPCR superfamily, they share similar structures, which are used to classify the GPCRs into six main classes (A – F) (6, 7). Rhodopsin-like Class A is the largest class, accounting for around 90% of GPCRs (8). Structurally, Rhodopsin-like GPCRs have a GpcrRhopsn4 domain, an eighth helix, and a palmitoylated cysteine at the C-terminal tail (9). Over 94% of pharmacological GPCR targets are Class A GPCRs (2, 3), emphasizing their potential for novel drug development.

To elicit cellular signaling, the activated GPCRs need to couple with intracellular transducers such as heterotrimeric G proteins, which are formed by Gα, Gβ, and Gγ subunits. Mammalian cells contain various G protein subunits that could combine to form diversified heterotrimeric G proteins, and each subunit, such as Gα, could transduce the signals independently (10). The G protein subunits regulate key effectors (adenylyl cyclases [ACs], guanylyl cyclases [GCs], phospholipase C [PLC], etc.) to generate the second messengers (cAMP, cGMP, Ca^2+^, inositol 1,4,5-triphosphate _[IP3]_, etc.), which in turn, trigger distinct signaling cascades (10). *Plasmodium* spp. share most of the functional characteristics of signaling pathways with mammalian cells. In particular, they use the same second messengers for the cAMP-protein kinase A (PKA) and cGMP-protein kinase G (PKG) signaling cascades (11, 12). However, because of the difference in primary sequences, GPCR-like proteins were discovered a decade ago in the P. falciparum genome, including the serpentine receptor 1 (SR1), SR10, SR12, and SR25 (13). Functional analysis revealed that SR10 regulates the duration of the intraerythrocytic development cycle (IDC) in both P. falciparum and P. chabaudi (14). PfSR25 acts as a K^+^ sensor in PLC activation, IP_3_ synthesis, and cytosolic Ca^2+^ mobilization (15). An IP_3_-responsive rise in cytosolic Ca^2+^ is essential for merozoites to release microneme-resident adhesion proteins during erythrocyte invasion (16). Furthermore, Ca^2+^-dependent signaling cascades are also involved in gametogenesis. When gametocytes are ingested by a mosquito, changes in the environment trigger cGMP synthesis and rise in cytosolic Ca^2+^, which subsequently activates calcium-dependent protein kinases (CDPKs). The CDPKs control DNA replication during the male gamete formation and protein synthesis required for life cycle progression in the mosquito (17, 18). Recently, a GPCR-like protein, 7-Helix-1, has been characterized in P. falciparum. Disruption of the *7-helix-1* gene leads to impaired gamete formation and reduced transmission of malaria parasites to mosquitoes (19). Since gametocytes are obligative for malaria transmission (20), identification of GPCR-like signal transducers in sexual stages may provide a promising target for the blockade of malaria transmission.

Here, we identified a conserved Class A GPCR subfamily member GPR180 in *Plasmodium* and studied its function during sexual development in P. berghei. Although PbGPR180 is highly expressed in gametocytes, *pbgpr180* gene disruption did not affect gametocytogenesis but impaired gametogenesis and transmission to the mosquito. We also present evidence that PbGPR180 plays an essential role in relaying the gametogenesis-inducing signals. Furthermore, we show that the PvGPR180 ortholog in P. vivax could rescue the defects of *pbgpr180*-deficient P. berghei, suggesting the conservation of this signaling pathway in other *Plasmodium* parasites. Importantly, we showed that an anti-PbGPR180 monoclonal antibody (MAb) or anti-PvGPR180 serum reduced the transmission of the parasites to mosquitoes. Together, we identified *Plasmodium* GPR180 as an important signal transducer during gametogenesis and a potential target for developing drugs or vaccines to block malaria transmission.

## RESULTS

### *Plasmodium* GPR180 encodes a new class of GPCRs predominantly expressed in sexual stages.

To identify potential signal transducers during sexual development, we searched the parasite genomes for genes that are expressed in gametocytes and encode proteins with seven transmembrane domains. Bioinformatic analysis identified a *gpr180*-like gene in all *Plasmodium* species (Fig. S1A), with the GPCR-like transmembrane domain located in the C terminus, as predicted using the HMMER program (21). The ∼250 aa GPCR domain contains residues that are highly conserved in the Rhodopsin-like GPCR transmembrane domain (pfam10192: GpcrRhopsn4) (Fig. S1B) (22), which classifies the GPR180 proteins as Class A (Rhodopsin-like) family members of GPCRs (Fig. S1C). Comparison between the rodent parasites (P. berghei and P. chabaudi) and human parasites (P. falciparum and P. vivax) also revealed >79% identity in the GpcrRhopsn4 domain of the GPR180 orthologs (Fig. S1D).

**FIG 1:**
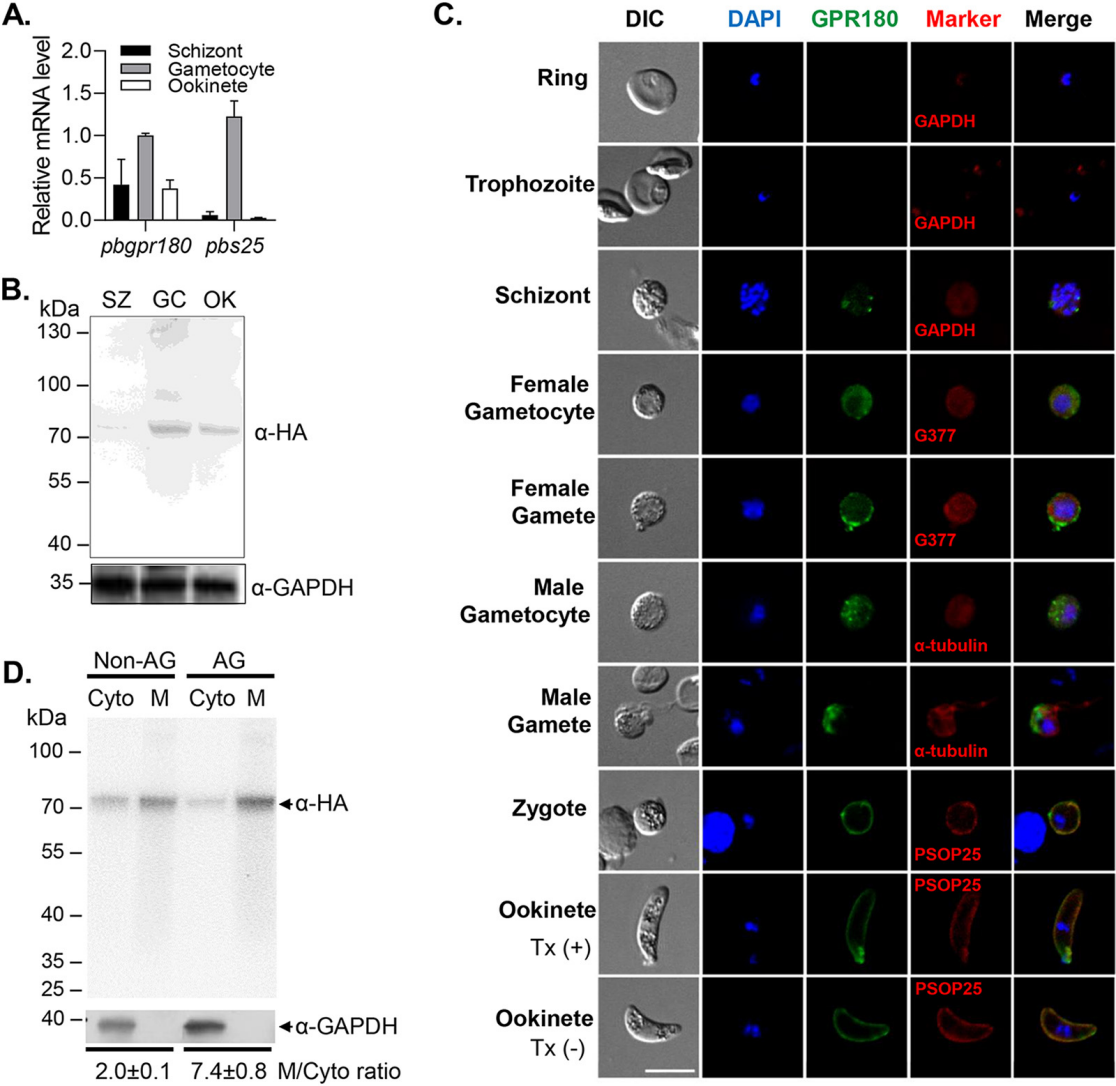
The transcription, expression, and subcellular location of PbGPR180. (A) The relative transcript level of *pbgpr180* in schizonts, gametocytes and ookinetes of P. berghei determined by real-time RT-PCR analysis. The *β-actin* gene was used for internal control. (B) Expression of PbGPR180HA in schizonts (SZ), gametocytes (GC), and ookinetes (OK) determined by Western blotting. The anti-HA MAb was used for detecting PbGPR180-HA (∼81 kDa). Equal loading was estimated using polyclonal rabbit anti-GAPDH antibodies (∼38 kDa). (C) The subcellular localization of the PbGPR180 protein during parasite development. Unless notified, all parasite stages were treated with 0.1% Triton X-100. The two ookinete images show membrane association of PbGPR180HA with (Tx [+]) or without (Tx [-]) Triton X-100 treatment. The differential interference contrast (DIC) images, the DAPI-stained nuclei (blue), PbGPR180-HA (green), and colocalization markers (red) of parasites are shown. Scale bar = 5 μm. (D) Cell fractionation analysis of PbGPR180-HA protein in nonactivated (non-AG) and activated gametocytes (AG). Western blot of the cytoplasmic (Cyto) and membrane (M) fractions of the gametocyte lysates was probed with the anti-HA antibody and the anti-GAPDH antibody (a marker for the cytoplasmic fraction), respectively. The M/Cyto ratio indicates the signal intensity ratio of the membrane/cytoplasmic fraction of the PbGPR180-HA in non-AG and AG, respectively.

We used the rodent malaria parasite P. berghei to study the function of GPR180 during development. We first determined PbGPR180 expression in purified schizonts, gametocytes, and ookinetes. Real-time PCR analysis detected the *pbgpr180* transcript in all the stages examined, but with about 2-fold higher abundance in gametocytes than other stages (Fig. 1A). To detect the PbGPR180 protein, we engineered a P. berghei transgenic line (PbGPR180-HA), where the C terminus of the endogenous *pbgpr180* gene was tagged with a 3×HA tag (Fig. S2A). After transfection, drug selection, and parasite cloning, we obtained parasite clones with correct 5′ and 3′ integration of the HA tag at the *pbgpr180* locus as verified by diagnostic PCR (Fig. S2B). The PbGPR180-HA clone 2 (C2) was further verified using primers flanking the engineered region and was selected for further analysis. Western blots of protein extracts from mixed parasite stages using the anti-HA MAb detected an ∼81 kDa protein band in the PbGPR180-HA parasite, but not in the wild-type (WT) parasite, consistent with the predicted size of the PbGPR180-HA fusion protein (Fig. S2C). Western blot further confirmed PbGPR180 expression in schizonts, gametocytes, and ookinetes, with gametocytes showing a much higher PbGPR180 level than other stages (Fig. 1B), consistent with the relative abundance of the *pbgpr180* transcript in different stages.

We next analyzed the expression and localization of PbGPR180-HA protein using an indirect immunofluorescence assay (IFA). We observed fluorescence in schizonts, gametocytes, gametes, and ookinetes, but not in the ring and trophozoite stages (Fig. 1C). In schizonts, male and female gametocytes, the PbGPR180 fluorescence displayed diffused and occasionally punctate staining in the cytoplasm of the parasites (Fig. 1C). However, more PbGPR180 fluorescence was associated with the plasma membrane in female gametes, whereas it was mainly detected at the residual body of exflagellating male gametocytes (Fig. 1C). In both zygote and ookinete, PbGPR180 protein was predominantly associated with the plasma membrane. Furthermore, it was detected in ookinetes under both permeabilizing and nonpermeabilizing conditions, suggesting surface localization (Fig. 1C and Fig. S3). This change in PbGPR180-HA localization during gametogenesis was further investigated by subcellular fraction analysis. Western blot analysis of the PbGPR180-HA protein in the membrane and cytoplasmic fractions showed that it was distributed at an ∼2:1 ratio in nonactivated gametocytes, but this ratio was increased to 7.4:1 in activated gametocytes (Fig. 1D). Consistent with the Western blot analysis, strong fluorescence was observed on gametes even without membrane permeabilization (Fig. S3). These results collectively indicate that gametocyte activation stimulates PbGPR180-HA redistribution from the cytoplasm to the plasma membrane.

### PbGPR180 is required for efficient gametogenesis.

To investigate the function of GPR180 in the life cycle of P. berghei, we generated two *pbgpr180* knockout (KO) lines Δ*pbgpr180*^Del^ (with the PbGEM-340051 plasmid) and Δ*pbgpr180*^KO^ (with the pL0034-PbGPR180-KO plasmid) using a double-crossover homologous recombination strategy (Fig. S4A, C). Successful deletion of the *pbgpr180* was confirmed by integration-specific PCR and Western blot analysis (Fig. S4B, D). Two independent clones, K1 of Δ*pbgpr180*^Del^ and K10 of Δ*pbgpr180*^KO^, were selected for further analysis. To determine whether *pbgpr180* KO affected parasite growth, we infected BALB/c mice with 1 × 10^6^ infected RBCs (iRBCs) of the WT parasite or Δ*pbgpr180* (clones K1 and K10) and monitored asexual parasitemia daily. We did not observe noticeable differences of asexual parasitemia between the WT-and Δ*pbgpr180*-infected mice (Fig. S4E). In addition, mice infected by the WT and Δ*pbgpr180* parasites also exhibited similar survival curves (Fig. S4F).

**FIG 2:**
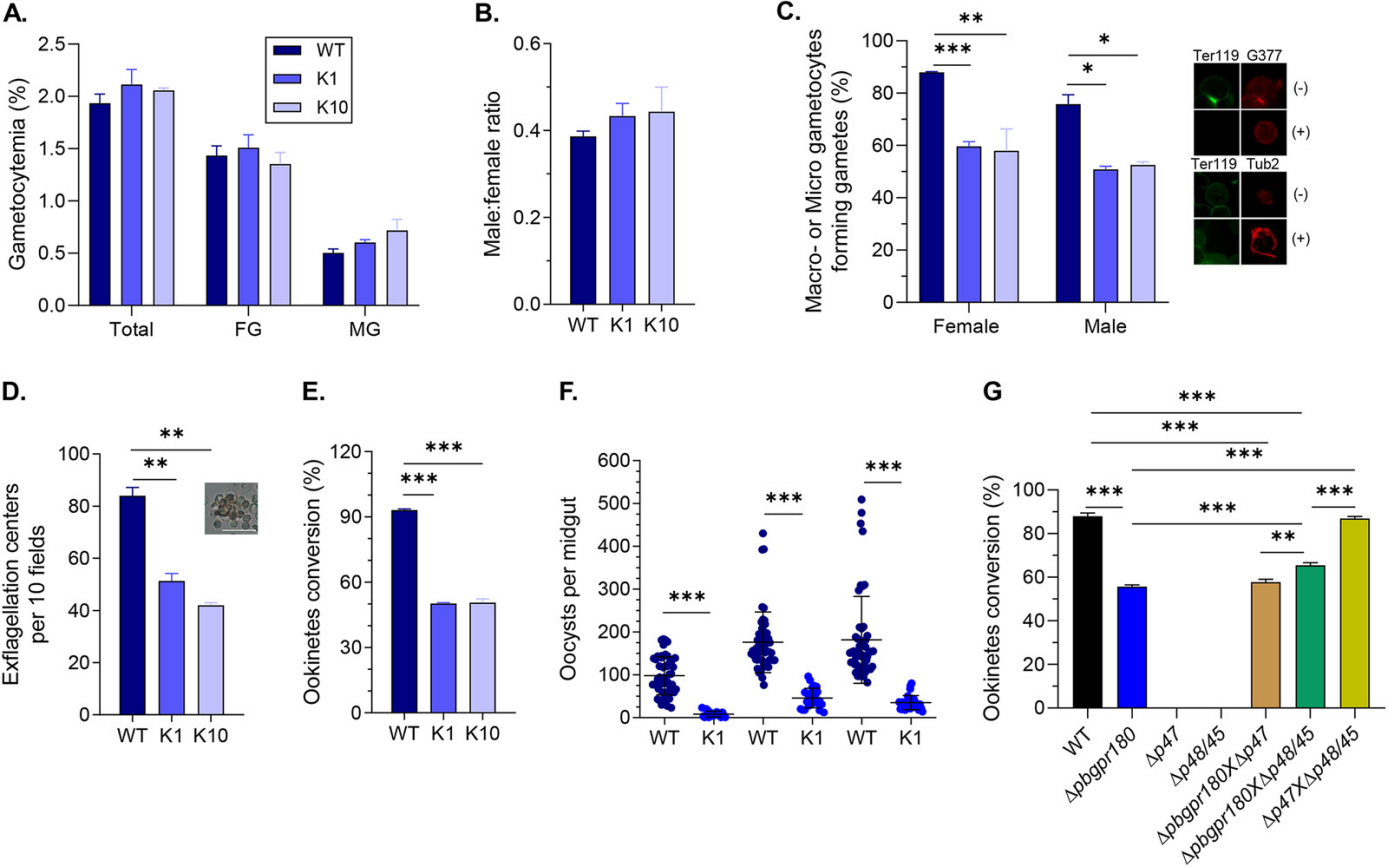
Phenotypes of the Δ*pbgpr180* parasites during sexual development. (A) Gametocytemia of the wild-type (WT) and Δ*pbgpr180* parasites (K1 and K10 clones) at 3 dpi. FG: female gametocyte. MG: male gametocyte. (B) Male/female gametocyte ratios of the WT and Δ*pbgpr180* parasites at 3 dpi. (C) The proportions of macrogametocytes or microgametocytes forming gametes (%) in WT and Δ*pbgpr180* parasites at 3 dpi. Representatives of gametocytes (-, Ter119 positive and α-tubulin II [Tub2]/G377 positive) and gametes (+, Ter119 negative and α-tubulin II [Tub2]/G377 positive) were shown on the right panel. (D) The number of exflagellation centers per 10 fields in the WT and Δ*pbgpr180* parasites. A representative image of the XA-stimulated exflagellation center under a light microscope is shown. Scale bar, 20 μm. (E) Ookinete conversion rate (%) in the WT and Δ*pbgpr180* parasites. The conversion rate is the percentage of Pbs21-positive parasites that had successfully differentiated into elongated ‘banana-shaped’ ookinetes. (F) Infection intensity of the *An. stephensi* mosquitoes feeding on the WT and Δ*pbgpr180* (K1) parasites. Mean oocyst numbers per experimental feed (±95% confidence interval) were determined from 50 mosquitoes on day 10 postfeeding from 3 independent experiments. (G) Ookinete conversion rates after crossing the Δ*pbgpr180* parasites with the female-defective (Δ*p47*) or male-defective (Δ*p45/48*) parasites. All bar graphs in this figure show Mean ± SD from three biological replicates were shown. *, *P* < 0.05; **, *P* < 0.01; ***, *P* < 0.001.

**FIG 3:**
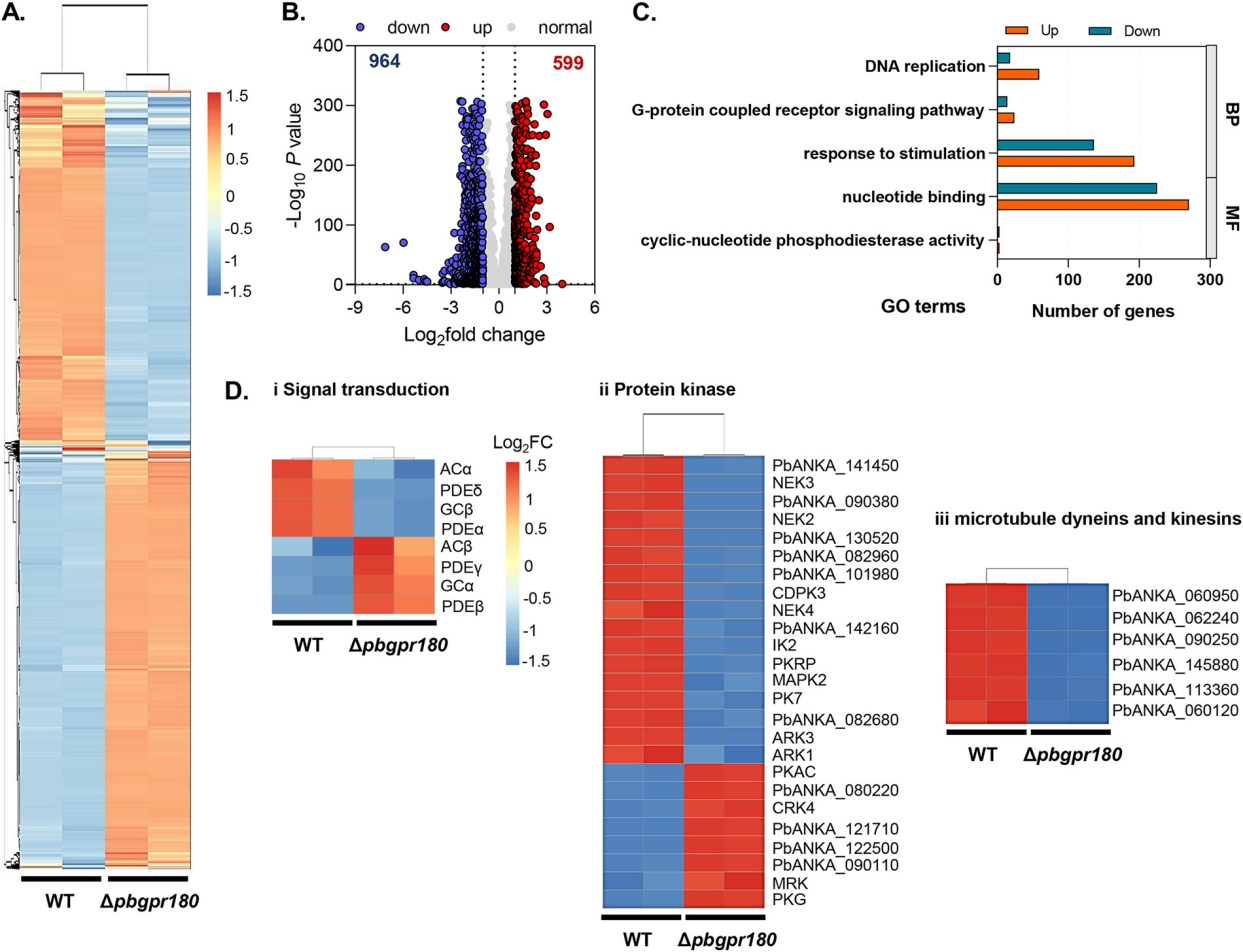
Transcriptome analysis for gametocyte stage of Δ*pbgpr180* parasites by RNA-seq. (A) Hierarchical clustering of all genes with significant changes in expression. The normalized FPKM values are shown on the vertical axis, and strain information (WT and Δ*pbgpr180*) on the horizontal axis. Clustering is based on Spearman correlation coefficients and plotted using an R program. Duplicates from each experimental group clustered independently (upper dendrogram). Refer to Table S2A for the data sets used to generate this figure. (B) Volcano plot showing the extent and significance of upregulated (red) and downregulated (blue) genes in the Δ*pbgpr180* parasites compared to WT (absolute Log_2-fold_ change > 1). Refer to Table S2B. (C) Gene ontology enrichment analysis of significantly 2-fold regulated genes in Δ*pbgpr180* parasites compared to WT parasites. GO terms representing the Biological Processes (BP) and Molecular Function (MF) are presented in blue (downregulated) and orange (upregulated) bars, respectively. Refer to Table S2C. (D) Heatmaps showing differential expression of selected genes in the WT and Δ*pbgpr180* parasites. Refer to Table S2D.

We next examined whether *pbgpr180* KO affected sexual development. *Pbgpr180* KO did not affect gametocytogenesis; the gametocytemia and sex ratio at 3 days postinfection (dpi) were similar between the WT and *pbgpr180* KO parasites (Fig. 2A and B). However, *pbgpr180* KO significantly affected gametogenesis. Gametes were distinguished from gametocytes based on the lack of the RBC membrane (negative for Ter119 staining), while female gametes were differentiated based on staining with the anti-Pbg377 antibodies. Compared to the WT parasites, the percentage of female gametocytes forming gametes in the two Δ*pbgpr180* clones K1 and K10 had a 28.3% and 30% reduction, respectively (Fig. 2C). Similarly, *pbgpr180* KO caused an ∼1.6-fold and ∼2-fold reduction in the number of exflagellation centers in the K1 and K10 clones, respectively (Fig. 3D and Fig. S5). These defects in gametogenesis ultimately led to reduced conversion of female gametocytes to ookinetes during *in vitro* culture. Whereas >93% of WT zygotes were transformed into mature ookinetes after 24 h of culture, Δ*pbgpr180* parasites had ∼50% conversion rate (Fig. 2E). In direct mosquito feeding assays, a 52% reduction in the percentage of infected mosquitoes was observed in those feeding on Δ*pbgpr180*-infected mice compared to the WT control (Fisher’s exact test, *P* < 0.001, Table 1). Meanwhile, mosquitoes feeding on Δ*pbgpr180*-infected mice exhibited an 80.5% decrease in oocyst density compared to the WT control (Fig. 2F).

**TABLE 1:** Transmission of Δ*pbgpr180* parasites in *An. stephensi*

	WT			Δ*pbgpr180*		
Parasite	M1	M2	M3	M1	M2	M3
Mosquito infected/dissected	49/50	50/50	50/50	18/50	25/50	28/50
Prevalence of infection (%)^*a*^	98	100	100	36	50	56
Mean prevalence (%)			99.3			47.3
Reduction in prevalence (%)^*b*^						52.0***
Oocyst intensity^*c*^	98.1	177. 7	183.0	8.3	47.5	33.4
SEM^*d*^	6.3	10.9	15.9	1.6	5.4	3.4
Mean oocyst intensity			152.9			29.7
Reduction in oocyst intensity (%)^*e*^						80.5 ***

a The prevalence of infection was calculated by the number of mosquitoes with oocysts/total mosquitoes dissected in each group × 100%.

b The percent reduction of prevalence was calculated as % mean prevalence_WT_ − % mean prevalence_Δ_*_pbgpr180_*; Fisher’s exact test; *** *P* < 0.001.

c Mean number of oocysts per mosquito midgut.

d Standard error of the mean.

e The percent reduction in oocyst intensity was calculated as (mean oocyst intensity_WT_ − mean oocyst intensity_Δ_*_pbgpr180_*)/mean oocyst intensity_WT_ × 100%; Mann-Whitney *U* test; *** *P* < 0.001.

To further determine whether the gametogenesis defect of the Δ*pbgpr180* parasites was sex-specific, we performed *in vitro* cross-fertilization experiments using parasite lines defective in either female gametes (Δ*pbs47*) or male gametes (Δ*pbs48*/*45*). As expected, neither the Δ*pbs47* nor Δ*pbs48*/*45* parasite line could form ookinetes, whereas mixing both parasite lines restored the ookinete conversion rate to a similar level as the WT parasite (Fig. 2G). However, while the Δ*pbgpr180* K1 parasite alone exhibited an ookinete conversion rate of 55.6%, co-incubation of Δ*pbgpr180* K1 with the Δ*pbs47* parasite only resulted in a slight, insignificant increase in ookinete conversion rate to 57.8% (*P* = 0.075). In comparison, co-incubation of Δ*pbgpr180* K1 with the Δ*pbs48*/*45* parasite increased the ookinete conversion rate to 65.5% (*P* < 0.001, Fig. 2G), suggesting a more severe defect of the Δ*pbgpr180* parasite in female gametes.

### *Pbgpr180* deletion leads to dysregulated expression of genes involved in the signaling pathway during sexual development.

To understand the molecular basis of the impaired gametogenesis due to *pbgpr180* KO, we compared the transcriptomes of activated gametocytes in the WT and Δ*pbgpr180* K1 parasites by RNA-seq analysis. The RNA integrity numbers for the WT and Δ*pbgpr180* K1 were 7.0 ± 0.3 and 6.8 ± 0.2, respectively. A total of 4435 transcripts were identified using a false discovery rate (FDR) of 5%, calculated based on the expected number of FPKM (Table S1A). The two replicates of the WT and the Δ*pbgpr180* parasites showed high within-group reproducibility, with the Pearson’s correlation coefficient reaching R^2^ = 0.998 and 0.999 between the two WT replicates and the two Δ*pbgpr180* replicates, respectively (Fig. 3A). Compared to the WT, 599 genes were upregulated, while 964 genes were downregulated (Log_2-fold_ change of > 1 and adjusted *P-*value < 0.05) (Fig. 3B and Table S1B).

Gene ontology (GO) enrichment analysis revealed that biological processes associated with these dysregulated transcripts include “DNA replication,” “G-protein-coupled receptor signaling pathway,” and “response to stimulation” (Fig. 3C and Table S1C). Notably, a wide range of dysregulated genes is assigned to molecular functions in “nucleotide binding” and “cyclic-nucleotide phosphodiesterase activity” (Fig. 3C and Table S1C). These dysregulated transcripts include genes encoding enzymes involved in the cyclic nucleotide signaling pathways, such as adenylyl cyclases (ACα and ACβ), guanylyl cyclases (GCα and GCβ), and phosphodiesterases (PDE) (α, β, γ, and δ), indicating that the initial steps of the cyclic guanosine 3’5′-monophosphate (cGMP)-protein kinase G (PKG)-Ca^2+^ signaling cascade were disturbed in the Δ*pbgpr180* parasites (Fig. 3D and Table S1C). In addition, we observed that the downstream components of the cGMP-PKG-Ca^2+^ signaling cascade, *nek3* and *mapk*2, were downregulated by 3.9 and 2.6-folds, respectively, in the Δ*pbgpr180* line (Fig. 3D and Table S1C). Also consistent with the extensive membrane remodeling and vesicular discharge during gametogenesis, we observed that genes associated with microtubule-based movement, including several kinesin and dynein family members, were significantly downregulated in the Δ*pbgpr180* parasite (Fig. 3D and Table S1D). Together, these data indicated that *pbgpr180* KO led to a dysregulated intracellular signaling network involved in gametogenesis in P. berghei.

### *Pbgpr180* deletion impairs the PKG-mediated signaling.

**FIG 4:**
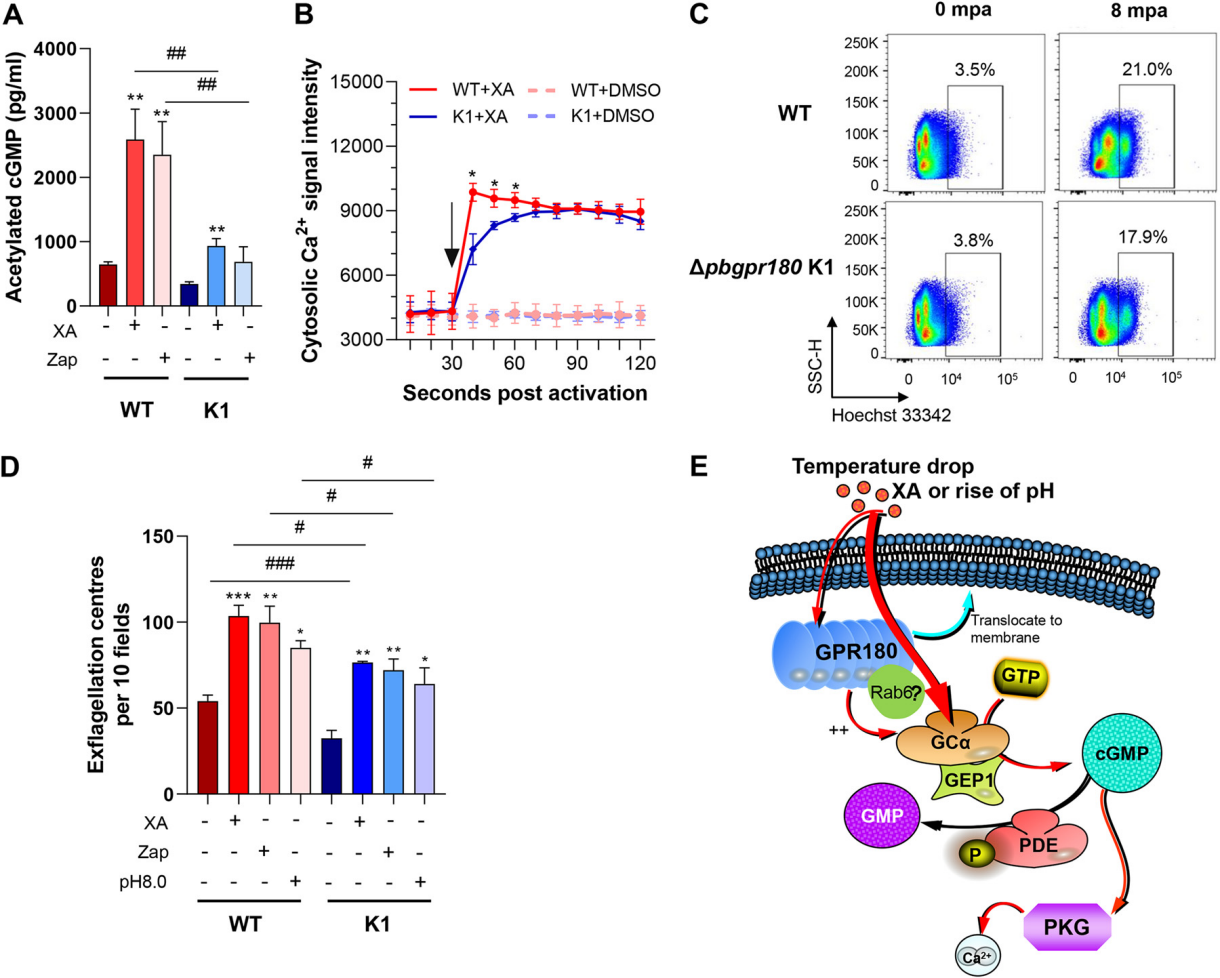
Effects of *pbgpr180* deletion on the cGMP-PKG-Ca^2+^ signaling pathway in gametogenesis. (A) Intracellular cGMP levels in the WT and Δ*pbgpr180* K1 gametocytes at the baseline and 2 min after adding 100 μM XA or 100 μM Zap at 25°C. (B) Flow cytometry analysis of cytosolic Ca^2+^ mobilization in XA-stimulated gametocytes using Fluo-8 probe. Purified gametocytes were preloaded with Fluo-8, and signals were collected 30 s before adding XA or DMSO. Black arrows indicate the time of XA or DMSO (solvent) addition. The data were normalized based upon starting levels of cytosolic Ca^2+^. (C) Flow cytometry analysis of genomic DNA replication in XA-stimulated gametocytes of the WT and Δ*pbgpr180* K1 parasites. The parasites were fixed with 4% paraformaldehyde at the indicated time post XA-stimulation, followed by staining with Hoechst 33342. mpa, minutes post activation. (D) Exflagellation center counts of the WT and Δ*pbgpr180* (K1) parasites after treatment with either XA (100 μM), or Zaprinast (Zap, 100 μM), or pH 8.0 at 25°C for 15 min. (E) A hypothetical model illustrating GPR180’s upstream location in the PKG-mediated signaling cascade during gametogenesis. *, *P* < 0.05; **, *P* < 0.01; and ***, *P* < 0.001 were for statistical comparison between treatment and control subgroups within the WT and Δ*pbgpr180* K1 (K1) groups (Student's *t* test). #, indicates *P* < 0.05; ##, *P* < 0.01; and ###, *P* < 0.001 (Student's *t* test) for comparison between the WT group and K1 group.

The dysregulation of several components in the PKG-mediated Ca^2+^ signaling cascade upon *pbgpr180* deletion prompted us to investigate if this pathway was affected during gametogenesis. First, we measured the intracellular cGMP synthesis during gametogenesis. The rise in cGMP levels is a crucial event during gametogenesis, as it stimulates PKG activity and leads to Ca^2+^ mobilization from the intracellular stores (23). It has been shown that the mosquito-derived gametocyte-activating factor xanthurenic acid (XA) activates GCα, resulting in a rapid elevation of cGMP levels (24, 25). The rise in cGMP levels can also be achieved by inhibiting the PDE activity using a specific inhibitor, zaprinast (Zap) (17, 26). As expected, XA or Zap treatment of the WT gametocytes resulted in significant increases in cGMP above the baseline level (Fig. 4A). Although XA or Zap treatment also triggered substantial increases of the cGMP levels in the Δ*pbgpr180* K1 gametocytes, the cGMP levels in the activated Δ*pbgpr180* K1 gametocytes were more than 2-fold lower than their corresponding levels in the WT gametocytes, suggesting that GPR180 functions upstream of the PKG signaling (Fig. 4A). We then followed the course of XA-stimulated Ca^2+^ mobilization in the activated gametocytes (25). Using Fluo-8 as a probe, we found that XA triggered a dramatic increase in cytosolic Ca^2+^ signal in the WT gametocytes, which reached the peak level at about 10 sec after treatment (Fig. 4B). Although we observed a similar trend in the rise of cytosolic Ca^2+^ signal in the Δ*pbgpr180* K1 gametocytes upon XA treatment, there was a slight delay in Ca^2+^ mobilization, with the Ca^2+^ signal intensity reaching the peak at 60 sec after XA treatment. In addition, between 10∼30 sec post XA-stimulation, the cytosolic Ca^2+^ signal intensity was significantly lower in the Δ*pbgpr180* K1 gametocytes than the WT gametocytes, suggesting that *pbgpr180* deletion affected both the speed and magnitude of Ca^2+^ mobilization during gametogenesis (Fig. 4B).

Male gametogenesis involves three rounds of genome replication, assembly of eight axonemes, and exflagellation. To detect whether *pbgpr180* deletion caused additional defects in genome replication, we analyzed the DNA content in gametocytes after XA induction by flow cytometry. At 8 min post XA-stimulation, WT gametocytes showed an increase of fluorescence signal from 3.5% to 21.0%, compared to the increase from 3.8% to 17.9% in Δ*pbgpr180* K1 gametocytes (Fig. 4C). Finally, we compared the effects of different environmental factors on exflagellation between WT and Δ*pbgpr180* K1 gametocytes. The exflagellation analysis was conducted in the exflagellation medium at 25°C under different inducing conditions (XA, Zap, or pH 8.0). Similar to the exflagellation analysis performed in mouse serum (Fig. 2D), XA treatment induced an ∼1.4-fold increase in the number of exflagellation centers in the WT than Δ*pbgpr180* K1 parasites (*P* < 0.001, Student's *t* test, Fig. 4D). In either the WT or Δ*pbgpr180* K1 gametocytes, Zap treatment induced a similar number of exflagellation centers as XA, confirming the bypassing of the requirement for the XA inducer (Fig. 4D). Moreover, a rise in pH from 7.4 to 8.0 at 25°C was also effective in inducing exflagellation. In either the WT or Δ*pbgpr180* K1 gametocytes, the number of exflagellation centers induced at pH 8.0 was only 10–15% lower than that from XA treatment (Fig. 4D). These results collectively indicate that the major defect in gametogenesis in Δ*pbgpr180* occurred at the step of cGMP synthesis, suggesting a possible function of GPR180 in relaying the external signals (Fig. 4E).

### PbGPR180 is associated with proteins of transporter functions.

To gain further insights into the PbGPR180 function, we performed immunoprecipitation (IP) using purified activated gametocytes of the PbGPR180HA parasite line with the MAb against HA. Precipitated proteins were identified by liquid chromatography and tandem mass spectrometry (LC-MS/MS). The same procedure was performed using WT P. berghei gametocytes as the negative control. A total of 36 proteins were identified in the IP from PbGPR180HA but not in the WT, each being represented by more than two unique peptides (Table S2A). If a cutoff threshold of GPR180HA/WT fold change of >2 and coefficient of variation (CV) < 0.1 was used, 66 proteins were enriched in the PbGPR180-HA IP (Table S2B). GO enrichment analysis of the putative PbGPR180-HA interactome revealed molecular function terms associated with *passive transmembrane transporter activity*, including a putative voltage-dependent anion-selective channel protein, EXP2, ATP synthase subunit gamma and beta, and Rab6 (Table S2C), implying that PbGPR180 may interplay with GTPase and membrane transporter proteins during gametogenesis.

### Allele replacement indicates functional conservation of GPR180 protein in *Plasmodium*.

With the presence of GPR180 orthologs in all sequenced *Plasmodium* genomes, we wanted to determine whether they are also functionally conserved during development. To do this, we replaced the *pbgpr180* coding sequence in P. berghei with the *pvgpr180* ortholog from the P. vivax Sal-I strain and simultaneously tagged the PvGPR180 with a 3×HA tag (Fig. S5A). Successful allele replacement was confirmed by diagnostic PCR (Fig. S5B), and PvGPR180-HA protein expression in two transgenic P. berghei parasite lines (R8 and R10) was confirmed by Western blotting using the anti-HA MAb (Fig. S5C). Compared to the WT P. berghei parasites, the two transgenic parasite lines showed similar asexual blood-stage growth in infected mice (Fig. S5D). Mice infected with the WT and transgenic parasites had similar survival curves (Fig. S5E).

**FIG 5:**
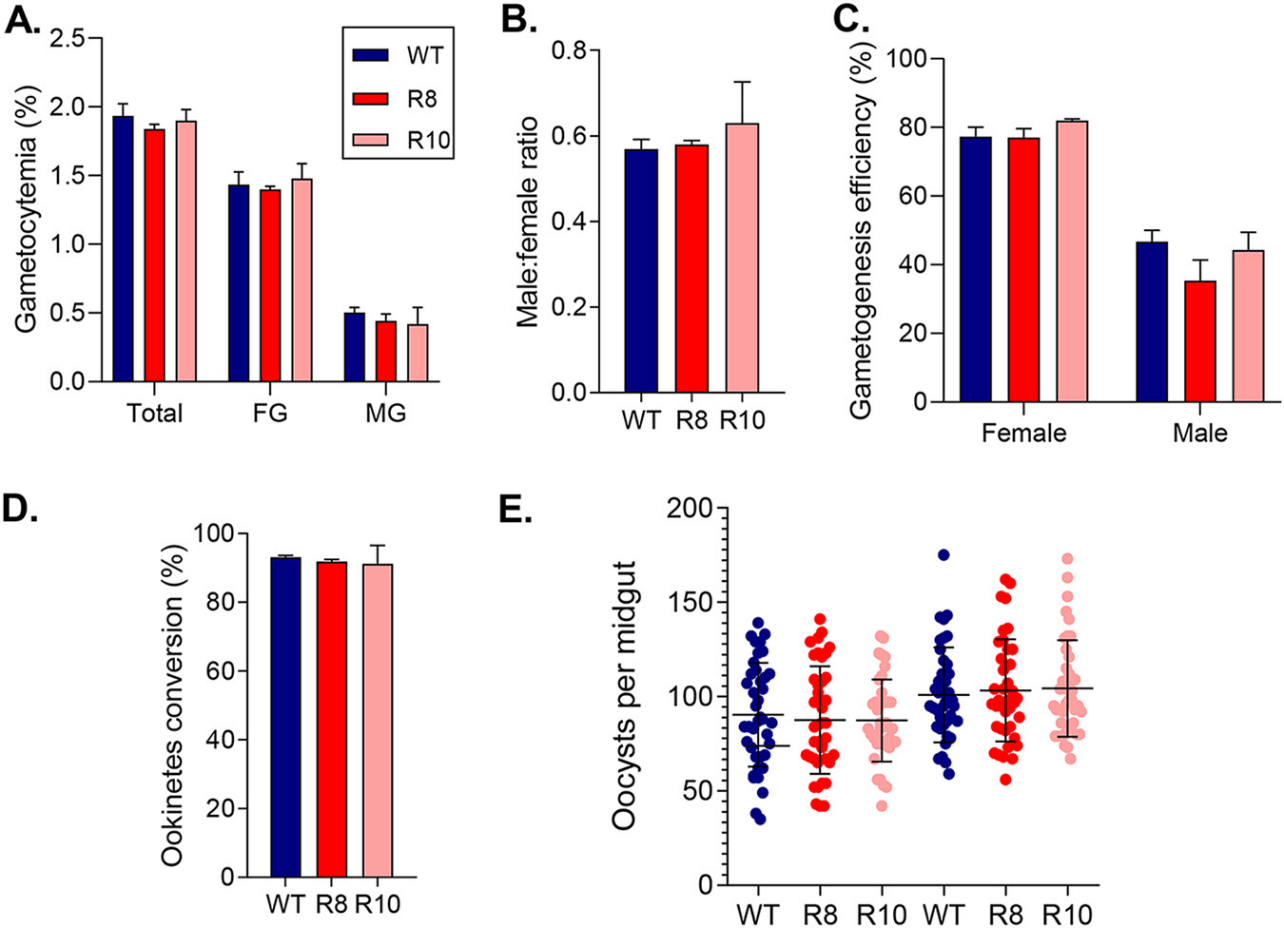
Phenotypes of the PvGPR180-transgenic parasites at the sexual stages. (A) Gametocytemia of WT and PvGPR180-transgenic (R8 and R10) parasites at 3 dpi. (B) Male/female gametocyte ratios of WT and PvGPR180-transgenic (R8 and R10) parasites at 3 dpi. (C) Gametogenesis efficiency (%) of the WT and PvGPR180-transgenic parasites at 3 dpi. (D) Ookinete conversion rates (%) of the WT and PvGPR180-transgenic parasites. (E) Infection intensity of the *An. stephensi* mosquitoes feeding on the WT and PvGPR180-transgenic parasites (R8 and R10). Each dot indicates the oocyst number of a mosquito, while the horizontal bars indicate the mean ± 95% CI. The graph shows two independent feeding experiments. For each feeding experiment, 40 mosquitoes were dissected for the WT and PvGPR180-transgenic (R8 and R10) parasites, respectively.

Next, we examined whether *Plasmodium* GPR180 is functionally conserved during sexual development. The two allele replacement clones had comparable levels of gametocytemia, sex ratio, and gametogenesis efficiency to the WT parasites (Fig. 5A–C). The transgenic parasites also displayed a similar efficiency (∼91%) in ookinete conversion to the WT parasite (93.1%) during *in vitro* ookinete culture (Fig. 5D). Finally, they showed similar levels of competence to infect *An. stephensi* mosquitoes as the WT parasite in a direct mosquito feeding assay. Specifically, both transgenic parasite lines and WT parasite resulted in 100% infection prevalence in fed mosquitoes. The oocyst density for the WT parasite (95.7/midgut) was also comparable with those for the R8 (95.3) and R10 parasites (95.9) (Fig. 5E). These findings demonstrate that PvGPR180 is functionally equivalent to PbGPR180 in the transgenic P. berghei.

### GPR180 is a potential target for blocking parasite transmission to the mosquito.

**FIG 6:**
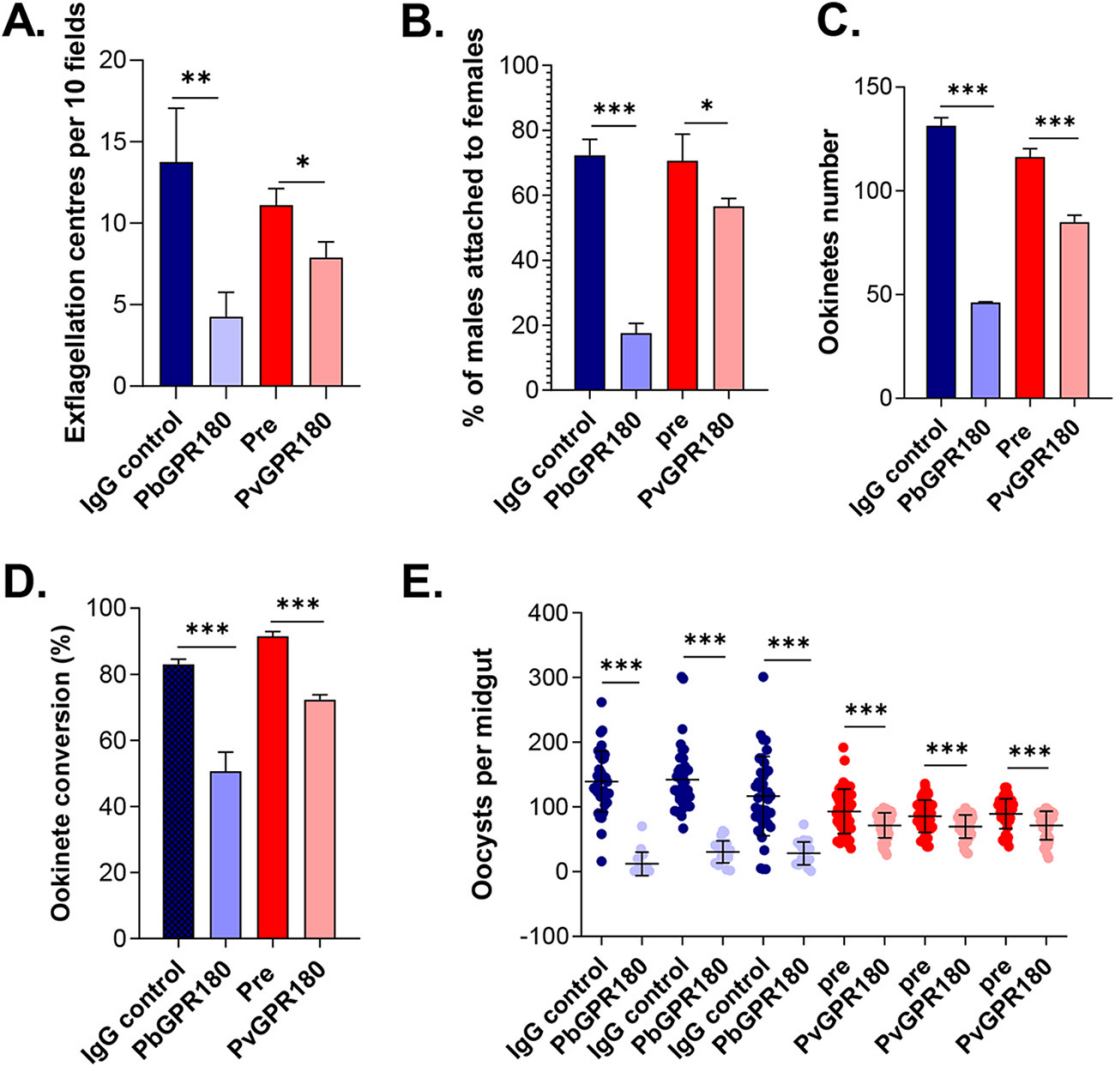
Transmission-blocking activity of the anti-PbGPR180 MAb and anti-PvGPR180 sera. The effect of anti-PbGPR180 MAb (0.2 μg/μL) or anti-PvGPR180 sera (1:5 dilution) on (A) exflagellation center formation, (B) interactions between male and female gametes, (C) ookinete formation, and (D) zygote-ookinete conversion from *in vitro* assays with the WT P. berghei and PvGPR180-R8 transgenic line, respectively. Error bars indicate mean ± SD (*n* = 4). (E) Direct mosquito feeding assay in mice infected with either the WT or the PvGPR180-R8 parasites (3 mice per group) and passively transferred with 10 μg anti-PbGPR180 MAb or 100 μL anti-PvGPR180 sera, respectively. Data points represent midgut oocyst numbers of individual mosquitoes in each group. Results from three independent experiments are shown. Error bars indicate mean ± SD (*n* = 3), expect for specifically indicated. Statistical significance was determined using Student's *t* test (A – D) or the Mann–Whitney *U* test (E). ns, not significant; *, *P* < 0.05; **, *P* < 0.01; ***, *P* < 0.001.

Given the surface location of GPR180 on ookinetes, we evaluated its transmission-blocking (TB) potential. We expressed a recombinant PbGPR180 fragment (150–370 aa) and a recombinant PvGPR180 fragment (26–390 aa) and produced a mouse MAb (4D3) and mouse sera against these recombinant proteins, respectively (Fig. S6). The PbGPR180 MAb and anti-PvGPR180 sera recognized their respective GPR180 proteins in the WT P. berghei parasite and the PvGPR180 allele replacement parasite lines R8 and R10 (Fig. S4D and S5C). From *in vitro* studies, the anti-PbGPR180 MAb (0.2 μg/μL) and anti-PvGPR180 sera (1:5 dilution) significantly inhibited the formation of exflagellation centers (Student's *t* test, *P* < 0.01), resulting in a 69.1% and 28.8% reduction compared to their respective control groups (Fig. 6A). Incubation with the anti-PbGPR180 MAb and anti-PvGPR180 sera also reduced the male-female gamete interactions by 9.5 (Student's *t* test, *P* < 0.001) and 1.2-folds (Student's *t* test, *P* < 0.05), respectively (Fig. 6B), implying decreased fertilization. Consequently, incubation with the MAb and sera led to a corresponding reduction in ookinete formation by 64.8% and 27.1%, respectively (Student's *t* test, *P* < 0.001, Fig. 6C). Furthermore, both the PbGPR180 MAb and PvGPR180 sera, when supplemented to *in vitro* ookinete cultures, significantly reduced ookinete conversion by 39.0% and 21.0%, respectively, compared to the controls (Student's *t* test, *P* < 0.001, Fig. 6D). Finally, we conducted mosquito feeding assays to determine the TB activity of the two antibodies. In a passive antibody transfer experiment, the anti-PbGPR180 MAb at 10 μg/mouse dramatically reduced mosquito infectivity, resulting in a 50% reduction in infection prevalence (Fisher’s exact test, *P* < 0.001, Table 2) and 81.9% in oocyst density (Mann–Whitney *U* test, *P* < 0.001, Fig. 6E), compared to the control groups. Although passively transferring 100 μL of anti-PvGPR180 sera did not result in a difference in mosquito infection prevalence from the control group (Table 2), it significantly decreased the oocysts intensity by 15.8% (Mann–Whitney *U* test, *P* < 0.001, Fig. 6E). These findings demonstrated that both the anti-PbGPR180 MAb and anti-PvGPR180 sera possessed evident TB activity.

**TABLE 2:** Transmission blocking activity of GPR180 MAb and anti-PvGPR180 sera in *An. stephensi*

Expt	Group	% Infected mosquitoes (infected/dissected)	% Reduction in prevalence^*a*^	Mean % reduction^*b*^	Oocyst density (mean ± SD)^*c*^	% Reduction in oocyst density^*d*^	Mean % reduction^*e*^
i	IgG control	92.5 (37/40)			139.2 ± 46.9		
	GPR180 mAb	45.0 (18/40)	47.5		12.1 ± 18.1	91.3	
ii	IgG control	100.0 (40/40)			142.3 ± 50.1		
	GPR180 mAb	50.0 (23/40)	57.5		30.5 ± 17.0	78.6	
iii	IgG control	97.5 (39/40)			116.7 ± 61.3		
	GPR180 mAb	52.5 (21/40)	45.0	50.0***	28.4 ± 17.8	75.7	81.9***
i	Pre	100 (50/50)			99.1 ± 33.8		
	PvGPR180	100 (50/50)	0		73.5 ± 17.3	25.8	
ii	Pre	100 (50/50)			82.6 ± 22.2		
	PvGPR180	100 (50/50)	0		72.5 ± 16.1	12.2	
iii	Pre	100 (50/50)			93.3 ± 22.5		
	PvGPR180	100 (50/50)	0	0	75.2 ± 20.7	19.4	15.8***

a % Reduction of prevalence = % prevalence_IgG control_ – % prevalence_GPR180 MAb_.

b Fisher’s exact test; ***, *P* < 0.001.

c The number of oocysts per midgut (mean ± SD).

d % Reduction in oocyst density = mean oocyst density_IgG control_ – mean oocyst density_GPR180 MAb_)/mean oocyst density_IgG control_ × 100%.

e Mann–Whitney *U* test; ***, *P* < 0.001.

## DISCUSSION

GPCRs are essential for numerous biological processes and are the main therapeutic targets in various diseases (2). To study how malaria parasites relay environmental signals, we characterized a GPCR, PbGPR180, which is highly conserved among *Plasmodium* spp., in P. berghei sexual development. Consistent with an earlier report from a genome-wide gene essentiality screening (27), *pbgpr180* is dispensable for asexual blood-stage development. However, *pbgpr180* KO led to considerable defects in gametogenesis for both male and female gametocytes, although the Δ*pbgpr180* parasite was not defective in gametocytogenesis. Interestingly, the stimulation of gametocytes to undergo gametogenesis was accompanied by redistribution in PbGPR180 from the cytoplasmic puncta to the cell periphery and increased association of PbGPR180 with the plasma membrane, a phenomenon similarly observed for two other signaling components in gametogenesis, GCα and the XA receptor GEP1, in P. yoelii and P. falciparum (24, 25). We provided experimental evidence that indicates the involvement of PbGPR180 in the cGMP-PKG-Ca^2+^ signaling pathway.

The cGMP-PKG-Ca^2+^ signaling pathway plays a central role in gametocyte activation and completion of gametogenesis (17, 28–30). The following findings support GPR180’s involvement in this pathway. First, transcriptome analysis showed that the dysregulated genes in the Δ*pbgpr180* parasite were significantly enriched in the GO term of cyclic nucleotide signaling pathways. Specifically, the mRNA levels of the major components in the cGMP-PKG-Ca^2+^ signaling cascade were altered considerably in Δ*pbgpr180* (31–33), including dysregulated GCα and GCβ, as well as upregulated PDEβ and PDEγ. Furthermore, *pbgpr180* KO caused dysregulation of several PKG-downstream components during gametogenesis, including the kinesin and dynein family members, which have been implicated to play a role in the microtubule-based movement of male gamete (34), and *nek3* and *mapk2*, which are required for axonemal beating in male gametes (35–37). These dysregulated signaling components and downstream effectors are consistent with the decreased efficiency in male and female gamete formation.

With the critical role of increased cGMP in gametocyte activation (38), its level must be kept in check in nonactivated gametocytes by the opposing activities of the GCs and PDEs (33). A recent study identified GCα as the main enzyme responsible for transducing the XA stimulation into elevated cGMP in gametocytes (25). Although the GCα transcript level was upregulated in Δ*pbgpr180* gametocytes, its activity might have been counteracted by the upregulated PDEβ and PDEγ, since the nonactivated Δ*pbgpr180* gametocytes had an almost 2-fold lower basal level of cGMP. Upon XA induction, the cytosolic cGMP level in the activated Δ*pbgpr180* gametocytes was also significantly lower (∼2.8-fold) than in the WT, suggesting either lower GCα or higher PDE activities or both in the Δ*pbgpr180* gametocytes. As a downstream event, the rapid mobilization of cytosolic Ca^2+^ due to elevated cGMP levels was also observed in the Δ*pbgpr180* gametocytes (39) but with a slight delay (lag phase 50 s after stimulation) and reduced magnitude after XA stimulation, which are likely attributed to the lower cGMP level after XA stimulation. These results indicate that this downstream Ca^2+^ mobilization step in the Δ*pbgpr180* gametocytes is functional, which may also explain the remaining exflagellation ability of Δ*pbgpr180* gametocytes. Interestingly, we also observed dysregulation of ACs in activated Δ*pbgpr180* gametocytes. The intracellular cAMP level is regulated by the activities of ACs and PDEs, and disruption of this balance in P. falciparum increases the rigidity of stage V gametocyte-iRBCs, and consequently block transmission of malaria (40, 41). Whether PbGPR180 protein participates in fine-tuning cAMP levels will be worthy of further investigations.

The baseline level of cGMP in nonactivated gametocytes is probably maintained by the PDE, since the inhibition of PDE activity by a specific inhibitor, Zap, can result in an uprise in cGMP to a sufficiently high level that induces gametogenesis in the absence of XA (17, 26). The Zap-elicited rise in cGMP and exflagellation was similarly observed in the Δ*pbgpr180* gametocytes, and the extents were similar to XA induction. The cGMP levels in Zap-treated Δ*pbgpr180* gametocytes were much lower than in WT gametocytes but proportional to the baseline cGMP level, suggesting lower GCα activity in Δ*pbgpr180* gametocytes. These results are consistent with the fact that the Δ*pbgpr180* gametocytes remained partially responsive to XA stimulation, but the full activity of the GEP1-GCα complex in response to XA requires PbGPR180. This would put PbGPR180 upstream of the PKG-mediated Ca^2+^ signaling cascade, acting as a sensor of environmental signals or facilitator of the GEP1 activity.

GPCRs play essential roles in regulating a cell’s response to environmental signals in eukaryotes (10). Our study indicates that PbGPR180 fulfills a similar function in signal transduction during gametogenesis. In the canonical GPCR-coupled pathway, ligand binding to a receptor leads to a conformational change in the receptor, which stimulates the association of a heterotrimeric G-protein (composed of α, β, and γ subunits) to the GPCR and the exchange of GDP for GTP in the Gα-subunit (42). The activated Gα-subunit dissociates from the Gβγ dimer, relaying the signals to downstream effectors like GCs. Malaria parasites lack canonical G-proteins and may use small GTPases for signal transduction (43). Interestingly, we identified a GTPase, Rab6, in the PbGPR180 interactome, a protein involved in vesicular trafficking from the Golgi to the endoplasmic reticulum (44, 45). However, whether Rab6 has any role in signaling and how PbGPR180 is connected to the cGMP-PKG-Ca^2+^ pathway remains undetermined.

Gametogenesis in malaria parasites is initiated once gametocytes are ingested by a mosquito and exposed to multiple environmental signals, such as a drop in temperature, a rise in pH, or the mosquito-derived XA (18, 23). The exact signal(s) that PbGPR180 responds to is still unknown. Comparable to previous reports, we found that XA, Zap, or pH rise could not overcome the low-temperature requirement for triggering exflagellation in neither WT nor Δ*pbgpr180* parasites (data not shown), supporting that a temperature decrease is essential for exflagellation. With a temperature drop to 25°C, XA- or Zap-stimulation could elevate the cGMP concentration over the threshold level needed to elicit downstream gametogenesis events, but the stimulated cGMP synthesis in the Δ*pbgpr180* gametocytes was substantially compromised, resulting in impaired exflagellation (33, 46). Based on these results, we propose a model for GPR180-mediated cGMP signaling in XA-stimulated gametogenesis. GPR180 may act as a sensor of extracellular signals of gametocyte activation (temperature drop, XA, or pH rise) and may regulate cGMP synthesis by modifying the activity of GCα-GEP1, probably through small GTPases such as rab6. Further work is needed to elucidate the position of the GPR180 in the signaling pathway of gametogenesis and the mechanisms by which it senses and relays environmental signals.

Since the primary transmission-blocking vaccine candidates include several membrane proteins expressed in gametes, zygotes, and ookinetes (47–50), we also tested the TB potential of GPR180 using both the WT P. berghei and a transgenic parasite expressing the GPR180 of the human malaria parasite P. vivax. Using both *in vitro* ookinete culture and direct mosquito feeding assays, we showed that the anti-GPR180 antibodies possessed obvious TB activity. Compared with the prefertilization TBV targets P48/45 (51), P230 (52), and HAP2 (53), as well as postfertilization targets such as P25/28 (50) and PSOP25 (49), GPR180 is expressed on both gametes and ookinetes and may elicit both pre- and postfertilization TB immunity, which encourages future studies in human malaria parasites. However, similar to previously identified TBV candidates, the GPR180 antigen only induced incomplete blocking of malaria transmission (∼70% with the MAb). Since TB activity can vary greatly among antibodies that bind different regions or epitopes of the target proteins, such as Pfs47, Pfs230, and PIMMS43 (54–56), future studies should explore other regions of GPR180 protein. In addition, its combination with other TBV candidates as a multitarget TBV may also increase TB activity.

In conclusion, this study identified GPR180 as an integral constituent of the signaling pathway during the gametogenesis of malaria parasites. The successful replacement of *pbgpr180* with *pvgpr180* suggests functional conservation of the GPR180 proteins in *Plasmodium* spp. Antibodies against the N-terminal fragments of GPR180 exhibited TB activity, supporting future efforts to refine *Plasmodium* GPR180 as a target for disrupting malaria transmission.

## MATERIALS AND METHODS

### Bioinformatics.

The *pbgpr180* genomic sequence (PlasmoDB ID: PbANKA_142930) was retrieved from PlasmoDB (https://www.plasmodb.org). Putative signal peptide, GpcrRhopsn4 domain, and transmembrane domain were predicted by SignalP v5.0 (http://www.cbs.dtu.dk/services/SignalP/), SMART (http://smart.embl-heidelberg.de/) and TMHMM v2.0 (http://www.cbs.dtu.dk/services/TMHMM-2.0/), respectively. The schematic presentation of the PbGPR180 protein was drawn by IBS 1.0.3 software (57). Multiple sequence alignment of GpcrRhopsn4 domain in GPR proteins was performed using the RPS-BLAST program of NCBI conserved domains search (https://www.ncbi.nlm.nih.gov/Structure/cdd/wrpsb.cgi). Sequence identity was calculated by using the Clustal Omega program (http://www.uniprot.org/align/). Phylogenetic analyses of the GPR180 orthologs were performed using the MEGA X and Eolview v3 software (58, 59).

### Parasite and mosquito maintenance.

Plasmodium berghei ANKA 2.34 strain and the Δ*pbgpr180* lines were maintained in 6–8-week-old Kunming outbred mice (Beijing Animal Institute, China) using standard protocols (60). The 6–8-week-old female BALB/c mice were used for phenotype analysis (Beijing Animal Institute, China). Parasitemia was monitored using Giemsa-stained tail blood smears (61). Phenylhydrazinium chloride (6 mg/mL in phosphate-buffered saline – PBS) was used to treat mice intraperitoneally (i.p.) at 200 μL/mice 3 days before infection to induce hyper-reticulosis. All animal experiments were carried out in compliance with the guidelines of the animal ethics committee of China Medical University. Female *An. stephensi* Hor strain mosquitoes were reared at 25°C and 75% humidity with a 12-h light/dark cycle on 10% (wt/vol) glucose solution under standard laboratory conditions (60). For mosquito transmission experiments, blood feeding of female mosquitoes was performed on anesthetized BALB/c mice at 3 dpi.

### Plasmid construction, parasite transfection, cloning and diagnostic PCR.

To tag the endogenous *pbgpr180* with a 3×HA tag, an 1140 bp (nucleotide positions [nt] 938–2077 bp) was amplified from the P. berghei genomic DNA (gDNA) and ligated in the ApaI and *Sac*II sites of the pL0034 plasmid as the 5′ homologous region (5R). Then, the 871 bp of the *pbgpr180* 3′-untranslated region (UTR) (nt +1–+871 bp) was amplified and inserted between XhoI and NotI as the 3′ homologous region (3R) to yield the final plasmid pL0034-PbGPR180-3×HA. For *pbgpr180* gene disruption, two plasmids were used. The PbGEM-340051 plasmid (to delete nt: 392 – 2080 bp of *pbgpr180* open reading frame–ORF) was obtained from PlasmoGEM (27, 62). To delete the entire *pbgpr180* ORF, an 1157 bp fragment containing the 5’UTR (nt: −1157 – −1 bp) and the 871 bp 3R described above were amplified and cloned into the HindIII/ApaI and XhoI/NotI sites, respectively, of the pL0034 vector to generate the plasmid pL0034-PbGPR180-KO. To replace the endogenous *pbgpr180* with the *pvgpr180* ORF, an 1157 bp fragment containing the 5’UTR of *pbgpr180* (nt: −1157 bp – −1 bp), the 2485 bp *pvgpr180* (PlasmoDB ID: PVX_123365) ORF, and the 871 bp 3R were amplified and cloned into HindIII/ApaI, ApaI/*Sac*II, and XhoI/NotI sites of the pL0034 vector, respectively, to generate pL0034-PvGPR180-R. The primers used for amplification are listed in Table S3.

For transfection, the pL0034-PbGPR180-3×HA, pL0034-PbGPR180-KO, and pL0034-PvGPR180-R plasmids were linearized with BglI and NotI, while the PbGEM-340051 plasmid was linearized with NotI, followed by ethanol precipitation. The linearized plasmid (15 μg) was electroporated into purified matured schizonts using the Nucleofector II (Lonza, Basel, Switzerland) (63). After transfection, the complete parasite suspension was injected intravenously via the tail vein into mice. Following a 24 h of incubation period, infected mice were treated for 7 days with pyrimethamine (Sigma-Aldrich, St. Louis, USA) via drinking water (70 μg/mL). Infected blood was collected, and correct integration was determined by integration-specific PCR (Table S3). Single clones of the parasites with stable construct integration were obtained by limiting dilution. Two parasite clones with correct integration for each transgenic line were selected for phenotypic analysis.

### Purification of P. berghei stages.

At 3 dpi, heparinized blood was collected from infected Kunming outbred mice (Beijing Animal Institute, China), and the parasites were cultured at 37°C overnight in the blood-stage culture medium consisting of RPMI 1640 with 50 mg/L penicillin, 50 mg/L streptomycin, 20% (vol/vol) heat-inactivated fetal calf serum (FCS). The culture was then fractionated on a 55% Nycodenz cushion to collect schizont stage parasites. For gametocytes purification, P. berghei-infected mice were treated with 20 mg/L sulfadiazine (Sigma-Aldrich) for 48 h to eliminate asexual stage parasites. Then, parasites were harvested and separated on a 48% Nycodenz/RPMI 1640 culture medium gradient (63, 64). For ookinetes, infected blood was harvested, cultured in a complete ookinete culture medium (RPMI 1640, 50 mg/L penicillin, 50 mg/L streptomycin, 20% [vol/vol] FCS, 6 U/mL heparin, pH 8.0) at 19°C for 24 h, and separated by 62% Nycodenz (48).

### Real-time reverse transcriptase (RT)-PCR analysis.

Total RNA was prepared in two independent experiments from the purified schizonts, gametocytes and ookinetes of P. berghei using the QIAamp RNA Blood minikit (Qiagen, Dusseldorf, Germany). Following the DNA eraser treatment to remove contaminating gDNA, 500 ng RNA was used for cDNA synthesis using the PrimeScript RT reagent kit (TaKaRa, Dalian, China). Real-time RT-PCR of *pbgpr180* and *pbs25* was performed using the TB Green Premix *Ex Taq* II (TaKaRa) with parasite 18S rRNA as a normalization standard (Table S1) (65). Control PCR was carried out without the addition of RT. The reactions were performed in ABI 7300 system (Applied Biosystems, Foster City, USA) using the following program: initial denaturation at 95°C for 5 min, followed by 40 cycles of 95°C for 15 s and 60°C for 1 min. Three technical replicates were performed for each biological experiment, and the fold change was calculated using the ΔΔC_t_ method (66).

### Indirect immunofluorescence assay (IFA).

IFA was carried out on different stages of the PbGPR180HA parasite as previously described (67). Briefly, 100 μL parasites-infected tail blood was collected into a 1.5 mL tube and washed three times with PBS. Parasites were fixed with 4% paraformaldehyde and 0.0075% EM grade glutaraldehyde in PBS for 30 min. Fixed parasites were washed once with PBS and then permeabilized with 0.1% Triton X-100 for 5 min on ice or nonpermeabilized, followed by PBS washing. After rinsing with 0.1 mg/mL of sodium borohydride (NaBH_4_) in PBS for 10 min, the preparations were blocked with 5% skimmed milk for 60 min at 37°C. After further washing with PBS for three times, parasites were incubated with the mouse anti-HA MAb (1:1000, Abcam, Cambridge, UK), rabbit anti-GAPDH antibodies (1:500, Abcam), or rabbit anti-α-tubulin II sera (1:500, a marker for male gametocytes/gametes), or rabbit anti-Pbg377 sera (1:500, a marker for female gametocytes/gametes), or rabbit anti-PbSOP25 sera (1:500) (48) at 37°C for 90 min. After three washes with PBS, the slides were incubated with Alexa Fluor-488 goat anti-mouse IgG or Alexa Fluor-594 anti-rabbit IgG antibody (1:500, Invitrogen, Carlsbad, USA) at 37°C for 30 min. Parasite nuclei were stained with 1 μg/mL of DAPI (Invitrogen). Parasites were then washed three times with PBS and mounted with ProLong Gold anti-fade reagent (Invitrogen), settled on a slide, covered by coverslips and visualized by Nikon C2 fluorescence confocal laser scanning microscope (Nikon, Tokyo, Japan). Images were captured and analyzed with the Image J software.

### Western blot analysis.

Protein concentrations in the lysates of purified schizonts, gametocytes, and ookinetes were quantified using the BCA kit (TaKaRa). The expression of PbGPR180 protein was compared by Western blotting using the mouse anti-HA MAb (1:2000, Abcam) and detected by horseradish peroxidase-conjugated goat anti-mouse IgG (H+L) antibodies (ThermoFisher) diluted at 1:25,000 in Tris-buffered saline with 0.1% Tween 20 (TBS-T). Proteins on the blot were visualized with the SuperSignal West Pico PLUS Chemiluminescent Substrate (ThermoFisher) on Tanon 4200 (Tanon, Shanghai, China). The relative molecular masses of proteins were estimated using the PageRuler Prestained Protein Ladder (ThermoFisher). The rabbit anti-GAPDH antibodies or the mouse anti-Hsp70 antibody was used for controlling protein loading.

Subcellular fraction analysis was performed using the Subcellular Protein Fractionation kit for cultured cells (ThermoFisher). Briefly, 1 × 10^8^ P. berghei gametocytes purified on 48% Nycodenz were either nonactivated or activated by incubation in the ookinete culture medium at 25°C for 15 min. Parasites were then resuspended in 200 μL of cytoplasmic extraction buffer and incubated on ice for 10 min with gentle mixing. The lysates were centrifuged at 10,000 *g* for 5 min at 4°C. The supernatant was centrifuged at 10,000 *g* for 10 min to obtain the cytoplasmic fraction. The pellet was resuspended in 200 μL of ice-cold membrane extraction buffer containing 1% [vol/vol] protease-inhibitor cocktail (ThermoFisher), vortexed for 5 sec, and incubated on ice for 10 min with gentle mixing. The homogenate was centrifuged at 10,000 *g* for 10 min to obtain the membrane fraction. Protein extracts were resolved by 10% SDS-PAGE and detected by immunoblotting using the anti-HA MAb and anti-GAPDH antibody as a cytoplasmic protein control. The protein band intensity was quantified using Image J.

### Phenotypic analysis.

To study the PbGPR180 functions during the IDC, five mice were injected, each with 1 × 10^6^ Δ*pbgpr180* parasites (clone K1 and K10), or the transgenic parasites expressing PvGPR180 (PvGPR180-R8 and -R10), or the WT P. berghei parasites. Parasitemia and mortality of the infected mice were monitored daily.

To study sexual stage development, mice were pretreated with 6 mg/mL phenylhydrazine and 2 days later infected with 1 × 10^6^ iRBCs. At 3 dpi, gametocytemia (mature gametocytes per 1000 RBCs) and male to female gametocyte ratio were determined using Giemsa-stained smears (68). For gametocyte egress analysis, drops of blood were collected and incubated with the exflagellation medium (RPMI 1640, 50 mg/L penicillin, 50 mg/L streptomycin, 20% [vol/vol] FCS, 6 U/mL heparin, pH 7.4, containing 100 μM XA) at 25°C for 30 min, and the male and female gametocytes were stained with anti-α tubulin II/FITC-conjugated Ter-119 or anti-Pbg377/FITC-conjugated Ter-119 antibodies, respectively. Male or female gametocytes were specified by positive α-tubulin II or Pbg377 fluorescence signals. They were considered not egressed if the signals for FITC-conjugated Ter119 staining were positive. For exflagellation center formation analysis, 10 μL of gametocyte-infected blood was obtained from the tail vein (the total number of gametocytes in 10 μL infected tail blood was adjusted to equal numbers between each group) and mixed immediately with 40 μL of exflagellation medium. The mixture was placed under a Vaseline-coated coverslip at 25°C, and 15 min later, exflagellation centers were counted over the next 10 min under a phase-contrast microscope at 40 ×. To evaluate the effects of the XA, Zap and pH rise on exflagellation, gametocytes were incubated in the exflagellation medium containing either 100 μM XA, or Zap (MCE, NJ, USA), or at pH 8.0 for 15 min at 25°C, and the ECs were counted as described above.

To determine *in vitro* ookinete formation, 10 μL infected blood containing an equal number of mature gametocytes was drawn into 90 μL ookinete culture medium and incubated at 19°C for 24 h. Cultured ookinetes were labeled with a mouse anti-Pbs21 antibody (1:500), and the number of ookinetes in 1 μL of the ookinete culture was counted under a fluorescence microscope. Cross-fertilization studies were performed with either *pbs47* knockout (Δ*p47*) or the *pbs48/45* (Δ*p48/45*) parasite lines generated earlier (68).

For direct mosquito feeding assay, *An. stephensi* mosquitoes were fed on WT- or Δ*pbgpr180*-infected mice at 3 dpi as previously described (69). In three replicated experiments, the prevalence of infection and oocyst numbers per midgut in 50 mosquitoes were determined on day 10 postfeeding.

### Flow cytometry analysis.

The DNA content in XA-stimulated gametocytes was measured as previously described (25). Briefly, mice were pretreated with phenylhydrazine 2 days before parasite infection. From 3 dpi, the infected mice were treated with sulfadiazine at 20 mg/L in drinking water for 48 h to remove asexual stage parasites. At 5 dpi, the WT and Δ*pbgpr180* gametocytes were purified on a 48% Nycodenz gradient as described above. Half of the purified gametocytes were immediately fixed with 4% paraformaldehyde, and the other half was incubated in an exflagellation medium for 8 min at 25°C before paraformaldehyde fixation. The samples were then washed in PBS three times and stained with Hoechst 33342 (ThermoFisher) for 30 min at 37°C. The fluorescence signal was quantified by the FACS Celesta flow cytometer and analyzed using the FlowJo 10 software (BD Pharmingen).

For Ca^2+^ mobilization analysis, purified WT and Δ*pbgpr180* gametocytes were maintained in the gametocyte maintenance buffer (GMB, 137 mM NaCl, 4 mM KCl, 1 mM CaCl_2_, 20 mM glucose, 20 mM HEPES, 4 mM NaHCO_3_, pH 7.24–7.29, 0.1% BSA) at 20°C with purity examined by Giemsa stained thin blood smear. The measurement of Ca^2+^ was performed as previously described (25). Briefly, the enriched gametocytes were washed three times with a Ca^2+^ free buffer (CFB, 137 mM NaCl, 4 mM KCl, 20 mM glucose, 20 mM HEPES, 4 mM NaHCO_3_, pH 7.2–7.3, 0.1% BSA) and loaded with 5 μM Fluo-8 (ATT Bioquest, CA, USA) in the CFB buffer for 20 min at 37°C. The labeled gametocytes were then washed twice with CFB and suspended in RPMI 1640 for flow cytometer analysis. The fluorescence signals of Fluo-8 were consecutively collected on a FACS Celesta flow cytometer from 30 sec before and 90 sec after the addition of XA (100 μM) and analyzed by the FlowJo 10 software (BD Pharmingen).

### Detection of cellular cGMP.

To measure cytosolic cGMP levels in WT and Δ*pbgpr180* gametocytes before and after XA stimulation, 1.5 × 10^7^ WT and Δ*pbgpr180* gametocytes were purified and maintained in GMB buffer on ice as described above. The samples were then treated with either 100 μM XA, 100 μM Zap, or DMSO for 2 min before being lysed with 0.2 M cold hydrochloric acid on ice for 10 min. The lysate was vortexed and passed through a 27G needle. The cGMP levels were analyzed using the cyclic cGMP enzyme immunoassay kit (Cayman Chemical, MI, USA).

### RNA sequencing.

Total RNA was extracted from the 48% Nycodenz purified activated gametocytes (incubation at 25°C for 15 min) of the WT and Δ*pbgpr180* K1 parasites using the Qiagen RNeasy kit (Qiagen, Dusseldorf, Germany). The purity of RNA was checked using the NanoPhotometer spectrophotometer (IMPLEN, CA, USA). RNA integrity was assessed using the RNA Nano 6000 assay kit of the Bioanalyzer 2100 system (Agilent Technologies, CA, USA). A total of 1 μg total RNA was used to purify the mRNAs using poly-T oligo-attached magnetic beads, followed by fragmentation using divalent cations under elevated temperature in the first-strand synthesis reaction buffer. First-strand cDNA was synthesized using random hexamer primer and RNase H. To select cDNA fragments of 100∼200 bp in length, the library fragments were purified with the AMPure XP system (Beckman Coulter, Beverly, USA). Adapters were ligated at 25°C for 10 min before PCR. PCR was performed with the Phusion High-Fidelity DNA polymerase, universal PCR primers, and the index (X) primer. PCR products were purified (AMPure XP system), and library quality was assessed on the Agilent Bioanalyzer 2100 system. The clustering of the index-coded samples was performed on a cBot Cluster Generation System using TruSeq PE Cluster kit v3-cBot-HS (Illumina, San Diego, USA). After cluster generation, the libraries were sequenced on an Illumina platform, and 150 bp paired-end reads were generated. Raw reads in the fastq format were first processed through in-house Perl scripts to remove low-quality reads, reads containing adapter, and ploy-N. Meanwhile, the Q20, Q30 and GC content of the clean data were calculated. The UMI (Unique Molecular Identifiers) was extracted by the UMI-tools (v2.0.4). Only clean UMI reads were kept for further analysis. RNA-seq reads from each sample were mapped to the P. berghei ANKA genome obtained from the NCBI reference genome (PbANKA01) using the Hisat2 (v2.0.4) (70). The UMI-tools (v1.0.0) were used to deduplicate reads based on the mapping coordinates and the UMI attached to the read. Cuffdiff v2.1 was used as the default method for normalization (71), while differential expression analysis was conducted using DESeq package (1.18.0) in R (72). The resulting *P* values were adjusted using Benjamini and Hochberg’s approach for controlling the FDR. Genes with an adjusted *P* value <0.05 found by DESeq were assigned as differentially expressed. GO enrichment analysis of differentially expressed genes was implemented by the GOseq R package, in which gene length bias was corrected. GO terms were considered significantly enriched by differentially expressed genes with a corrected *P*-value less than 0.05. The RNA-seq raw data were deposited in the NCBI via the GEO (accession number: GSE198287).

### Immunoprecipitation of HA-tagged PbGPR180 and mass spectrometry.

Activated gametocytes from the PbGPR180HA and WT P. berghei parasite lines were first lysed in 0.15% (wt/vol) saponin in PBS and harvested by centrifugation. Proteins were extracted using 10 cell pellet volumes of ice-cold RIPA lysis and extraction buffer containing the Halt phosphatase inhibitor cocktail (ThermoFisher). Samples were cleared by centrifugation at 130,000 × g for 5 min. Parasite lysates containing the HA-tagged PbGPR180 protein were added into Pierce Anti-HA Magnetic Beads (ThermoFisher) and incubated at room temperature for 30 min with mixing. The beads were collected with a magnetic stand, washed using the IP Lysis/Wash Buffer twice, and followed by washing once with ultrapure water. Finally, the beads were resuspended in 5 volumes of Elution Buffer, and the supernatants were collected, neutralized the low pH by adding neutralization buffer, and stored at −80°C for further analysis.

Eluted proteins were identified by LC-MS/MS. Proteins were run 4 mm into a 10% NuPAGE BisTris gel (ThermoFisher) and then excised. Proteins were reduced and alkylated prior to overnight trypsin digestion. The resulting digests were analyzed by LC-MS/MS using an Ultimate 3000 nanoRSLC HPLC, equipped with a 50-cm 75-m Acclaim Pepmap C18 column, coupled to an LTQ Orbitrap Velos Pro equipped with a Nanoflex electrospray source (all ThermoFisher). A gradient of 8% to 32% acetonitrile (pH 9.0), 0.1% formic acid over 60 min was used at a flow rate of 0.3l/min. The Orbitrap was operated in the data-dependent acquisition mode with a survey scan at 60,000 resolution and up to the 10 most intense ions selected for MS/MS. The resulting MS/MS data were processed using the Maxquant search engine (v.1.5.2.8). Tandem mass spectra were searched against the human UniProt database concatenated with reverse decoy database. Trypsin/P was specified as a cleavage enzyme allowing up to 4 missing cleavages. The mass tolerance for precursor ions was set as 20 ppm in the First search and 5 ppm in the Main search, and the mass tolerance for fragment ions was set as 0.02 Da. Carbamidomethyl on Cys was specified as fixed modification, and acetylation modification and oxidation on Met were specified as variable modifications. FDR was adjusted to < 1% and the minimum score for modified peptides was set > 40. The MS raw data were in submission to the ProteomeXchange Consortium via the PRIDE (accession number: PXD030837) (73).

For functional enrichment analysis, the identified proteins were classified by GO annotation into three categories: biological process, cellular compartment and molecular function. For each category, a two-tailed Fisher’s exact test was employed to test the enrichment of the differentially expressed protein against all identified proteins. The GO with a corrected *P*-value < 0.05 is considered significant. The Kyoto Encyclopedia of Genes and Genomes (KEGG) database was used to identify enriched pathways by a two-tailed Fisher’s exact test to test the enrichment of the differentially expressed protein against all identified proteins.

### Recombinant protein expression and antibody production.

The recombinant PbGPR180 protein (rPbGPR180, 150–370 aa) and PvGPR180 (rPvGPR180, PVX_123365, 26–390 aa) were produced as 6×His-tagged proteins using the yeast expression system (Genecreate Inc., Wuhan, China) and purified using a HisPur Ni-NTA Magnetic Beads (ThermoFisher, Waltham, USA). BALB/c mice were immunized three times with purified rPbGPR180 or rPvGPR180 protein. To produce the MAb against PbGPR180, the spleen cells of the immunized mice were extracted and fused with Sp2/0-Ag14 myeloma cells using the traditional polyethylene glycol method (74). The antibodies were screened by indirect antibody capture enzyme-linked immunosorbent assay as previously described (49). The IgG fractions were prepared by ammonium sulfate precipitation and purified on a Protein G column (ThermoFisher). The anti-PbGPR180 MAb clone 4D3 was selected for further analysis. The anti-PvGPR180 sera were raised by immunizing mice with the rPvGPR180 following a standard immunization protocol (60).

### Quantification of transmission-blocking activity of anti-GPR180 antibodies.

TB activities of the anti-PbGPR180 MAb and anti-PvGPR180 sera were estimated using both *in vitro* ookinete conversion and *in vivo* mosquito feeding assays (48, 61). In short, phenylhydrazine pretreated mice were injected with 1 × 10^6^ P. berghei iRBCs or the transgenic parasite R8. At 3 dpi, parasitemia was counted by Giemsa staining. The gametocyte activation, exflagellation, male/female gametes attachment, ookinete number and conversion experiments were performed as described above, except for that the ookinete culture medium was supplemented with the anti-PbGPR180 MAb or mouse IgG control (eBioscience, San Diego, USA), or the anti-PvGPR180 sera or pre-immune sera, respectively. Mosquito-feeding experiments were carried out as described previously (48). Briefly, three mice from each group were infected with the respective parasite lines. At 3 dpi, each mouse was passively transferred with either 10 μg anti-PbGPR180 MAb or 10 μg mouse IgG control, or 100 μL anti-PvGPR180 sera or 100 μL pre-immune sera one h before mosquitoes feeding. 10 days after the blood meal, the prevalence and intensity of infection in mosquitoes were determined in approximately 40 mosquitoes.

### Statistical analyses.

Data are presented as mean ± standard deviation (SD). Statistical differences of parasitemia, gametocytemia, exflagellation, gametocyte activation, male/female gamete interaction, ookinete numbers and conversion (%) between groups were analyzed using a two-tailed, unpaired Student's *t* test. The intensity of infection (oocysts/midgut) was analyzed using the Mann–Whitney *U* test, while infection prevalence (proportion of infected mosquitoes) was assessed using Fisher’s exact test. Survival curves were analyzed using a Kaplan–Meier’s survival test. *P* values less than 0.05 were regarded as statistically significant. Data analysis was performed using the GraphPad Prism 8.0.1 program (GraphPad, La Jolla, USA).

### Data availability.

The RNA-seq raw data were deposited in the NCBI via the GEO (accession number: GSE198287). The MS raw data were in submission to the ProteomeXchange Consortium via the PRIDE (accession number: PXD030837).
