# Supplementary material for: A G-Protein-Coupled Receptor Modulates Gametogenesis via PKG-Mediated Signaling Cascade in Plasmodium berghei
Source: Microbiol Spectr. 2022 Apr 11;10(2):e00150-22. doi: 10.1128/spectrum.00150-22 (PMC9045217; doi:10.1128/spectrum.00150-22)
Supplement: SUPPLEMENTAL FILE 1 — Supplemental material. Download spectrum.00150-22-s0001.pdf, PDF file, 1.3 MB [file spectrum.00150-22-s0001.pdf]

SUPPLEMENTAL MATERIAL

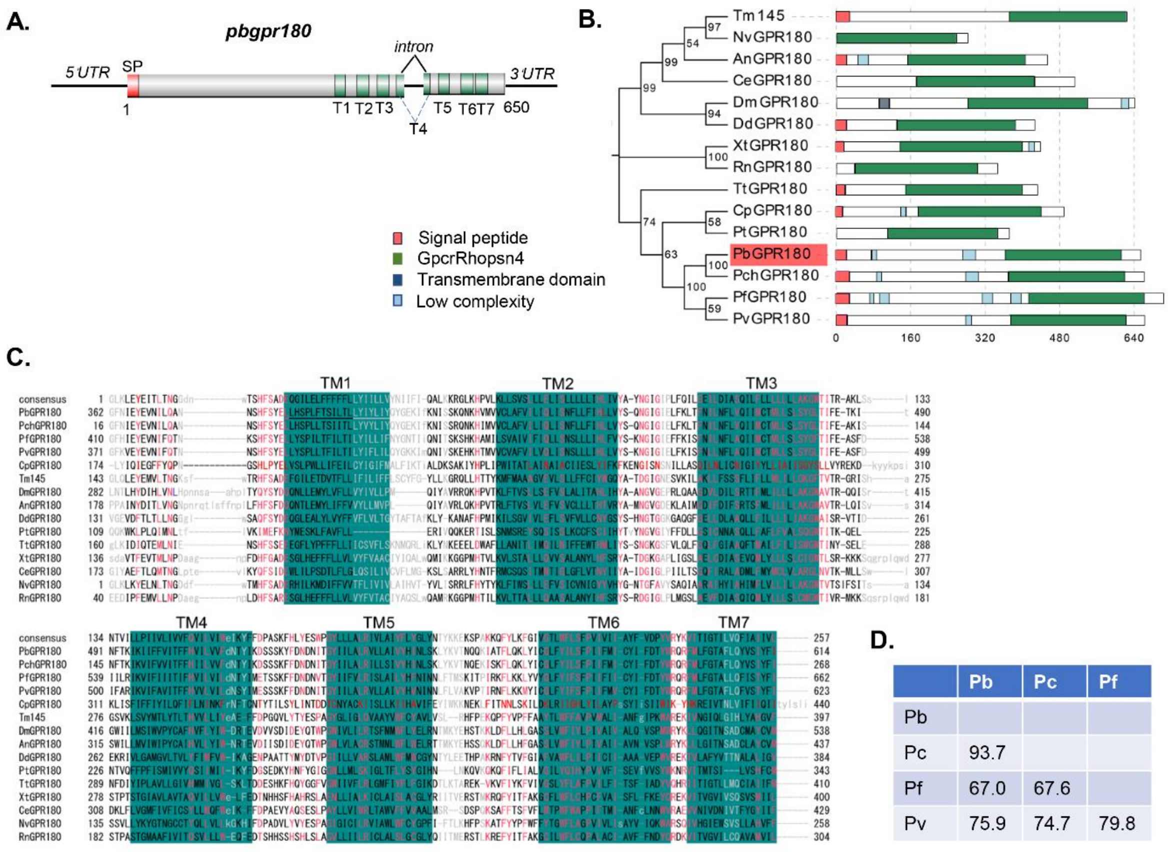

Fig S1. Bioinformatic analysis of GPR180. (A) Schematic illustration of *pbgpr180* gene in *P. berghei*. The red box indicates signal peptide (SP). The seven predicted transmembrane domains (T1-T7) are shown as green boxes. (**B)** A Neighbor-Joining tree of the GPR180 orthologs. Numbers beside each node indicate a percentage of 1000 bootstrap replications. GPR180 proteins include *P. berghei* GPR180 (PbGPR180, PlasmoDB ID: PbANKA_142930), *P. chabaudi* GPR180 (PchGPR180, PlasmoDB ID: PCHAS_143120), *P. falciparum* GPR180 (PfGPR180, PlasmoDB ID: PFL0655w), *P. vivax* GPR180 (PvGPR180, PlasmoDB ID: PVX_123365), *Cryptosporidium parvum* GPR180 (CpGPR180, CMU_008310), *Homo sapiens* (Tm145, GenBank no. NP_775904.2), *Drosophila melanogaster* GPR180 (DmGPR180, GenBank no. ABY21760.1), *Anopheles gambiae str. PEST* GPR180 (AnGPR180, GenBank no. XP_320314.4), *Dictyostelium discoideum* AX4 GPR180 (DdGPR180, GenBank no. XP_635150.1), *Paramecium tetraurelia* strain d4-2 GPR180 (PtGPR180, GenBank no.

XP_001442037.1), *Tetrahymena thermophila* SB210 GPR180 (TtGPR180, GenBank no. XP_001030210.2), *Xenopus tropicalis* GPR180 (XtGPR180, GenBank no. NP_001135514.1), *Caenorhabditis elegans* GPR180 (CeGPR180, GenBank no. NP_510017), *Nematostella vectensis* GPR180 (NvGPR180, GenBank no. XP_001627491.1), *Rattus norvegicus* GPR180 (RtGPR180, GenBank no. NP_001006995.1). (**C**) ClustalW alignment of the GpcrRhopsn4 domain of the GPR180 homologs. Conserved residues are shown in red color. The seven transmembrane domains (TM1 – 7) are highlighted in dark green color. (**D**) Sequence identity of the GpcrRhopsn4 domains between *Plasmodium* species. Pb (*P. berghei*), Pc (*P. chabaudi*), Pf (*P. falciparum*), and Pv (*P. vivax*).

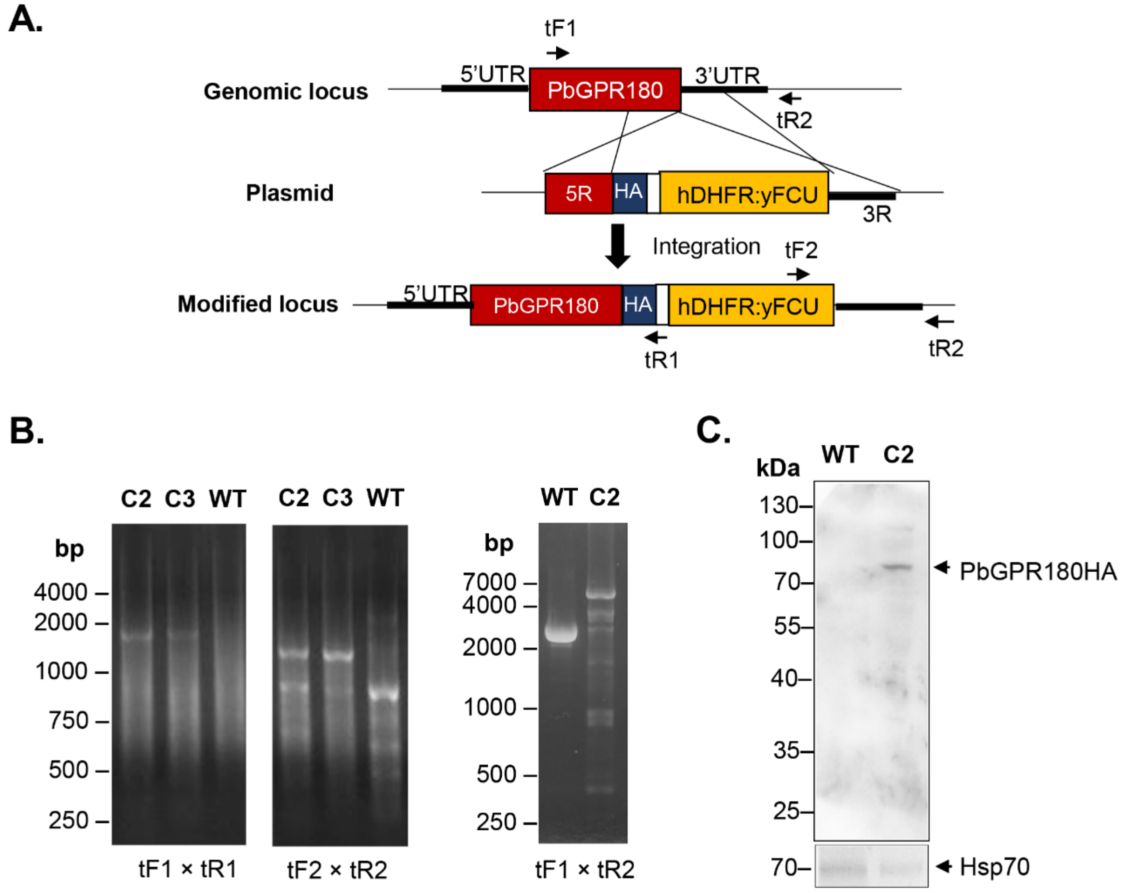

Fig S2. Tagging of the *pbgpr180* gene locus with a 3×HA tag in *P. berghei* ANKA parasite. (**A**) Schematic depicting of the strategy to generate the PbGPR180HA parasite. The

hDHFR:yFCU represents the human dihydrofolate reductase:yeast cytosine deaminase and uridyl-phosphoribosyltransferase expression cassette in the pL0034 plasmid, which positive selection by pyrimethamine and negative selection by 5-fluorouracil, respectively. Primers used for genotyping in (**B)** are indicated as arrows. (**B)** Genotyping PCR to confirm integration. PCR was performed using wild type (WT) *P. berghei* and the PbGPR180-HA parasite clone 2 (C2), and C3 genomic DNA as the template, respectively. The PCR product resulting from 5' integration with primers tF1×tR1 and 3' integration with primers tF2×tR2 is 1739 bp and 1485 bp, respectively. The PCR product resulting from the entire engineered region with primers tF1×tR2 is 2572 bp (WT) and 5448 bp (PbGPR180-HA C2), respectively. (**C)** Western blot confirming the tagging of PbGPR180 with the 3×HA tag. Parasite lysates of mixed stages were electrophoresed and probed with the anti-HA monoclonal antibody (upper panel) and anti-Hsp70 antibodies (lower panel) as a loading control. The arrow indicates the predicted band size (~81 kDa) of the recombinant PbGPR180HA protein.

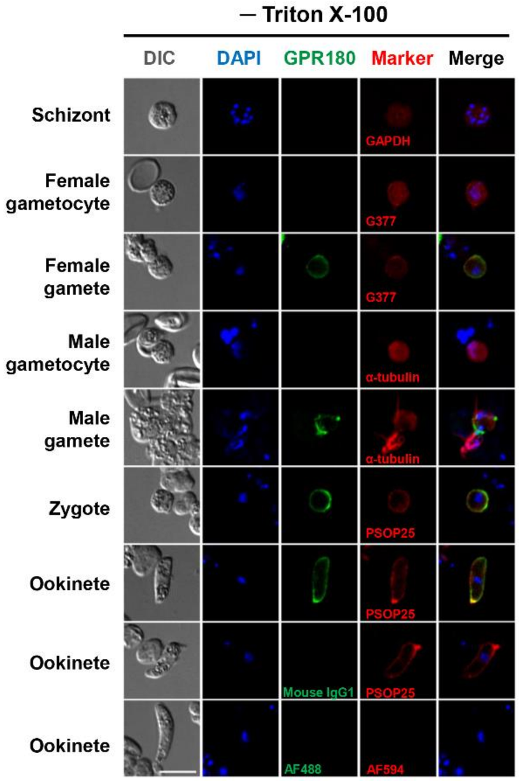

Fig S3. Localization of PbGPR180-HA protein by IFA without Triton X-100 permeabilization. The PbGP180HA parasites were co-labeled with the anti-HA monoclonal antibodies (mAb) and markers including GAPDH for asexual stages, Pbg377 (G377) for female gametocytes and gametes, α-tubulin II for male gametocytes and gametes, and PSOP25 for ookinetes. Alexa Fluor 488 (AF488)-conjugated goat anti-mouse IgG antibodies and Alexa Fluor 594 (AF594)-conjugated goat-anti-rabbit IgG antibodies were used as the secondary antibodies. PbGPR180HA ookinetes labeled with mouse IgG1 mAb and with the secondary antibodies only were used as negative controls. The differential interference contrast (DIC) images, the DAPI-stained nuclei (blue), PbGPR180-HA (green), and co-localization markers (red) of parasites are shown. Scale bar = 5 μm.

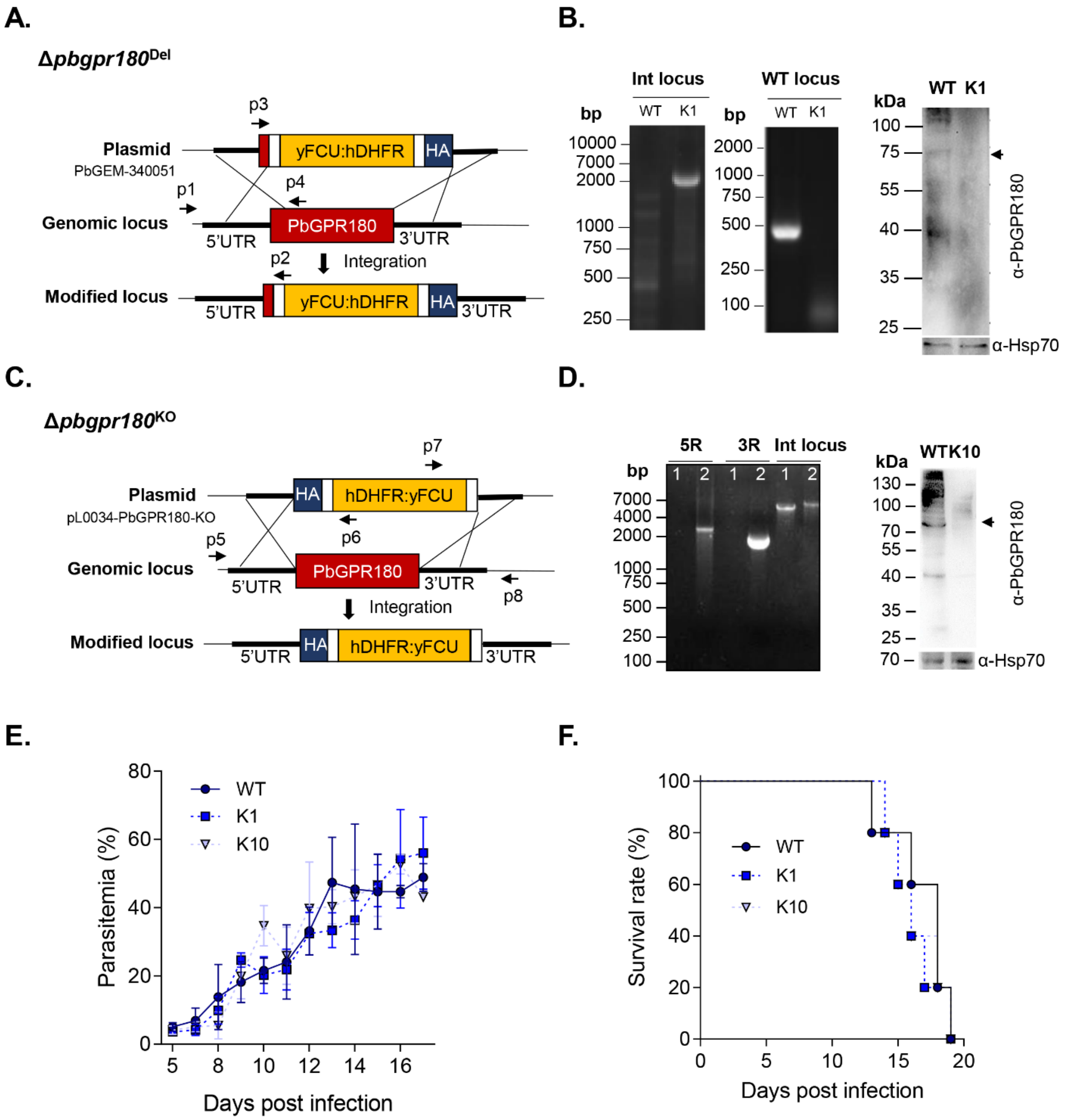

Fig S4. Knockout of *pbgpr180* gene in *P. berghei*. **(A)** Scheme depicting the generation of the *pbgpr180* gene nucleotide positions (nt) 392 – 2080 bp deletion (Del) parasite (Δ*pbgpr180*^{Del}). The yFCU:hDHFR box represents the yeast cytosine deaminase and uridyl-phosphoribosyltransferase: human dihydrofolate reductase expression cassette in the PbGEM-340051 plasmid, which positive

selection by pyrimethamine and negative selection by 5-fluorouracil, respectively. **(B)** Successfully editing of *pbgpr180* gene locus in Δ*pbgpr180*^{Del} clone K1 parasite was confirmed by genotyping PCR (left panel) and Western blot analysis (right panel), respectively. The integrate (Int) and wild type (WT) form of *pbgpr180* gene locus were detected by primers p1 × p2 (2116 bp) and primers p3 × p4 (486 bp) using wild-type *P. berghei* and Δ*pbgpr180* parasite genomic DNA as the template, respectively. For western blot analysis, parasite lysates prepared from mixed stages of WT and Δ*pbgpr180* ^{Del} K1 were probed with an anti-PbGPR180 mAb (upper panel), and anti-Hsp70 antibodies (lower panel) to monitor protein equal loading. **(C)** Scheme depicting the generation of the *pbgpr180* knockout (KO) parasite (Δ*pbgpr180*^{KO}). **(D)** Genotyping PCR (left panel) and Western blot (right panel) of parasite clones with modification in *pbgpr180* of *P. berghei*. Genotype PCR confirming *pbgpr180* KO in the Δ*pbgpr180* parasites. Lanes 1 and 2 used the wild-type *P. berghei* and Δ*pbgpr180* parasite genomic DNA as the template, respectively. 5' and 3' integration was verified using primers p5 × p6 (2218 bp) and p7 × p8 (1532 bp), respectively. The entire engineered locus of wild-type *P. berghei* and Δ*pbgpr180* parasite was verified using primers p5 × p8 (lane 1, 4509 bp; lane 2, 5317 bp). Western blot analysis of parasite lysates from wild wild-type *P. berghei* and Δ*pbgpr180*^{KO} clone K10 parasites were detected using anti-PbGPR180 mAb. The anti-Hsp70 (α-Hsp70) was used as a loading control. **(E)** Comparison of the proliferation curves of WT and the Δ*pbgpr180* parasites (clones K1 and K10). Parasitemia in parasite-infected mice was determined daily by light microscopy of Giemsa-stained blood smears. Data are the mean ± SD of three independent experiments. **(F)** The effects of *pbgpr180* disrupted on the survival of infected mice. Kaplan-Meier's survival curves of mice infected with the WT and Δ*pbgpr180* parasites. Each group had five mice. The graph was representative from three independent experiments.

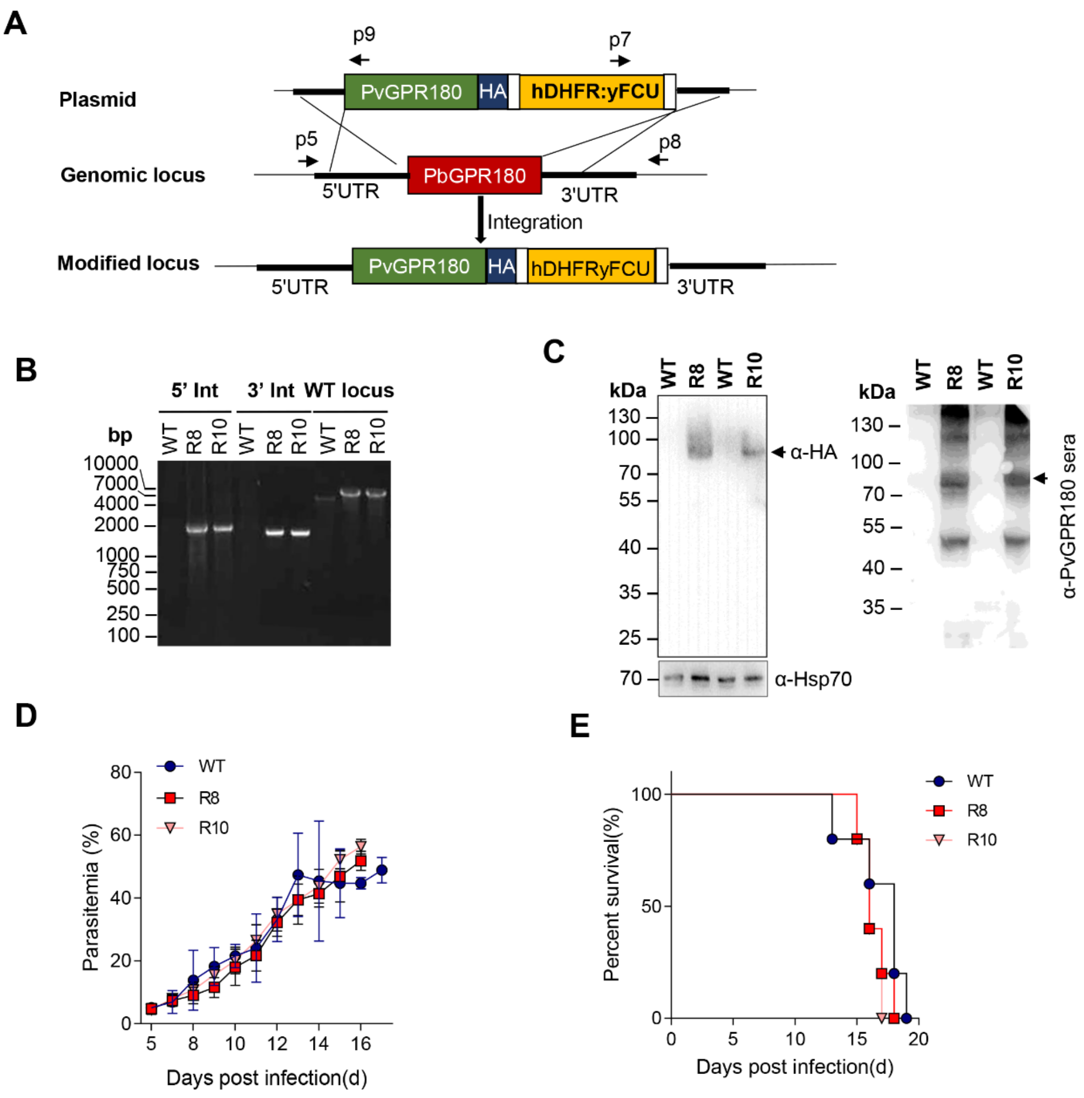

Fig S5. Replacement of *pbgpr180* in *P. berghei* with the *P. vivax* ortholog *pvgpr180*. (A) Schematic showing the replacement of the *pbgpr180* coding region with *pvgpr180* in *P. berghei* and simultaneous tagging of PvGPR180 with a 3 × HA tag. The primers used to verify the 5' integration are indicated by arrows. Thick lines indicate 5' and 3' UTRs. (**B)** PCR confirmation of integration of the *pvgpr180* coding sequence at the *pbgpr180* genomic locus. R8 and R10 are two transgenic parasite lines. 5' Int shows the PCR product to verify 5' integration (p5 × p9, 1760 bp), the 3' Int to verify 3' integration (p7 × p8, 1529 bp), and the WT locus shows the native and modified region (p5 × p8. native locus: 4509 bp; modified locus: 7754 bp). **(C)** Western blot confirmation of PvGPR180-R protein expression in PvGPR180-transgenic parasites

by using the anti-HA mAb (left) or anti-PvGPR180 sera (right). Hsp70 indicates approximately equal loading of the protein extracts. Arrow indicates recombinant PvGPR180 protein expressed in PvGPR180-R transgenic parasites. (**D**) Growth curves of the WT and transgenic parasites R8 and R10. Each mouse was inoculated with 1×10^6 iRBCs and parasitemia was monitored daily. Data are the mean ± SD of three independent experiments. (**E**) Kaplan-Meier's survival curve of mice infected with the WT and transgenic parasites. Each group has five mice. The graph is representative of three independent experiments.

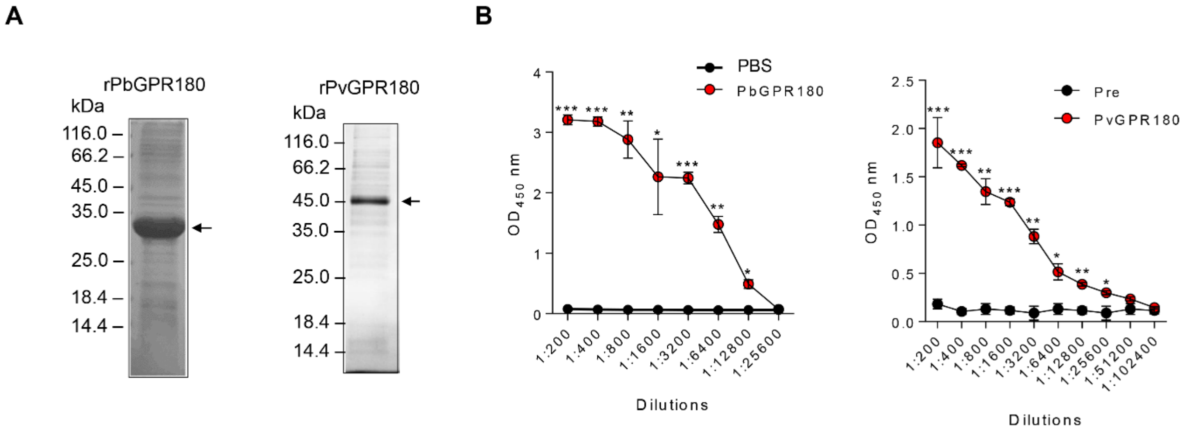

Fig S6. Purification of the recombinant PbGPR180 (rPbGPR180), rPvGPR180 protein and antibody response in mice immunized with rPbGPR180 and rPvGPR180 protein, respectively. (**A**) Purified recombinant rPbGPR180 (150–370 aa, ~30 kDa, left panel) and rPvGPR180 (26–390 aa, ~45 kDa, right panel) protein were subjected to electrophoresis on a 10% polyacrylamide gel and stained with Coomassie Brilliant Blue, respectively. Protein Ladder in kDa. (**B**) Female BALB/c mice were immunized with the rPbGPR180 and rPvGPR180 protein and adjuvants in PBS as a control. Total IgG titers after the final immunization were measured by ELISA with the plates coated with rPbGPR180 (left panel) and rPvGPR180 (right panel), respectively. Error bars indicate mean ± SD (n=3). *, $p < 0.05$, **, $p < 0.01$, ***, $p < 0.001$ represents the difference between rPbGPR180 or rPvGPR180 and PBS groups, respectively (Student's *t* test).
